# Arginine reprograms metabolism in liver cancer via RBM39

**DOI:** 10.1016/j.cell.2023.09.011

**Published:** 2023-11-09

**Authors:** Dirk Mossmann, Christoph Müller, Sujin Park, Brendan Ryback, Marco Colombi, Nathalie Ritter, Diana Weißenberger, Eva Dazert, Mairene Coto-Llerena, Sandro Nuciforo, Lauriane Blukacz, Caner Ercan, Veronica Jimenez, Salvatore Piscuoglio, Fatima Bosch, Luigi M. Terracciano, Uwe Sauer, Markus H. Heim, Michael N. Hall

**Affiliations:** 1 Biozentrum, University of Basel, 4056 Basel, Switzerland; 2 Institute of Molecular Systems Biology, ETH Zürich, 8093 Zürich, Switzerland; 3 Institute of Medical Genetics and Pathology, University Hospital Basel, 4031 Basel, Switzerland; 4 Department of Biomedicine, University of Basel, 4031 Basel, Switzerland; 5 Department of Biomedicine, Hepatology Laboratory, University and University Hospital Basel, 4031 Basel, Switzerland; 6 Department of Biochemistry and Molecular Biology, Universitat Autònoma de Barcelona, 08193 Barcelona, Spain; 7 Clarunis University Center for Gastrointestinal and Liver Diseases, 4031 Basel, Switzerland

**Keywords:** hepatocellular carcinoma, metabolism, arginine, ARG1, AGMAT, ASNS, RBM39, indisulam

## Abstract

Metabolic reprogramming is a hallmark of cancer. However, mechanisms underlying metabolic reprogramming and how altered metabolism in turn enhances tumorigenicity are poorly understood. Here, we report that arginine levels are elevated in murine and patient hepatocellular carcinoma (HCC), despite reduced expression of arginine synthesis genes. Tumor cells accumulate high levels of arginine due to increased uptake and reduced arginine-to-polyamine conversion. Importantly, the high levels of arginine promote tumor formation via further metabolic reprogramming, including changes in glucose, amino acid, nucleotide, and fatty acid metabolism. Mechanistically, arginine binds RNA-binding motif protein 39 (RBM39) to control expression of metabolic genes. RBM39-mediated upregulation of asparagine synthesis leads to enhanced arginine uptake, creating a positive feedback loop to sustain high arginine levels and oncogenic metabolism. Thus, arginine is a second messenger-like molecule that reprograms metabolism to promote tumor growth.

## Highlights

• Arginine is increased in liver cancer despite suppression of the urea cycle
• Increased uptake and decreased conversion to polyamines maintain high arginine
• Arginine binds the transcription regulator RBM39 to promote oncogenic metabolism
• RBM39 dependency in liver cancer could be exploited by molecular glue degraders

## Graphical abstract

**Figure:**
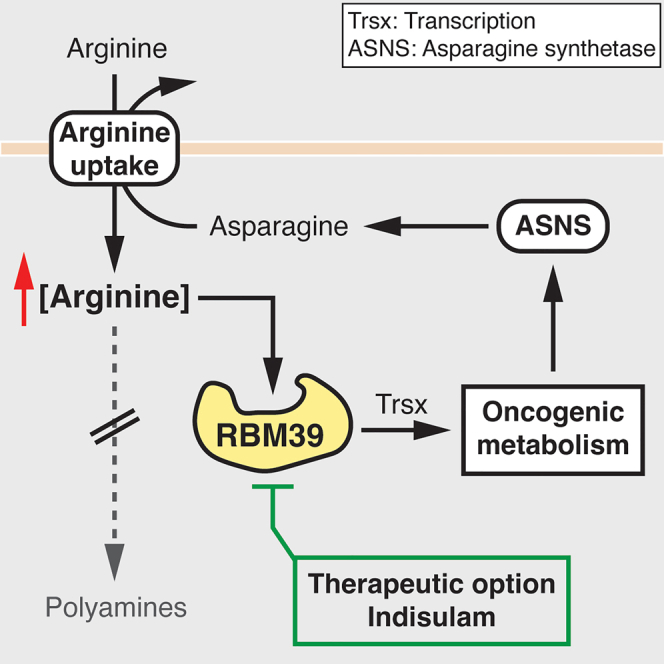

## Teaser

Arginine is a second messenger-like molecule that promotes liver tumorigenesis by binding to RBM39 and controlling metabolic gene expression. RBM39 depletion by the molecular glue degrader indisulam blocks pro-oncogenic metabolic reprogramming in hepatocellular carcinoma, suggesting that RBM39 is a therapeutic target in liver cancer.

## Introduction

Progress in the last decade has revealed that cancer is a metabolic disorder in which several, if not most, major metabolic pathways are rewired to enhance cell proliferation.^1^^,^^2^ The altered metabolic pathways include carbohydrate, amino acid, nucleotide, fatty acid, and lipid metabolism.^3^^,^^4^^,^^5^^,^^6^^,^^7^^,^^8^ However, the upstream mechanisms and downstream targets of metabolic reprogramming in cancer are largely unknown.

Rewiring of amino acid metabolism is common in cancer.^3^^,^^4^^,^^9^^,^^10^^,^^11^^,^^12^^,^^13^^,^^14^^,^^15^ For example, expression of argininosuccinate synthetase 1 (ASS1), the rate-limiting enzyme in arginine synthesis, is often altered in tumors. It is overexpressed in some cancers, including colon, lung, gastric, and ovarian cancer, but lost in other cancers, such as renal cell carcinoma (RCC), melanoma, prostate cancer, and hepatocellular carcinoma (HCC).^16^^,^^17^ However, despite considerable focus on ASS1, little is known about arginine levels in tumors.

Arginine is a highly versatile amino acid. Besides its role as a building block in protein synthesis, it is a precursor for polyamines, creatine, and nitric oxide. Arginine can also be interconverted with proline and glutamate and can promote cell growth by activating mTORC1.^3^ Furthermore, there is evidence that arginine impacts metabolism, at least in part, independently of mTORC1.^18^^,^^19^^,^^20^^,^^21^ We also note that arginine is produced by the urea cycle, of which ASS1 is a component. Given the above, we sought to investigate the role of arginine in HCC.

## Results

### Elevated arginine levels are necessary for liver tumorigenesis

**Figure 1:**
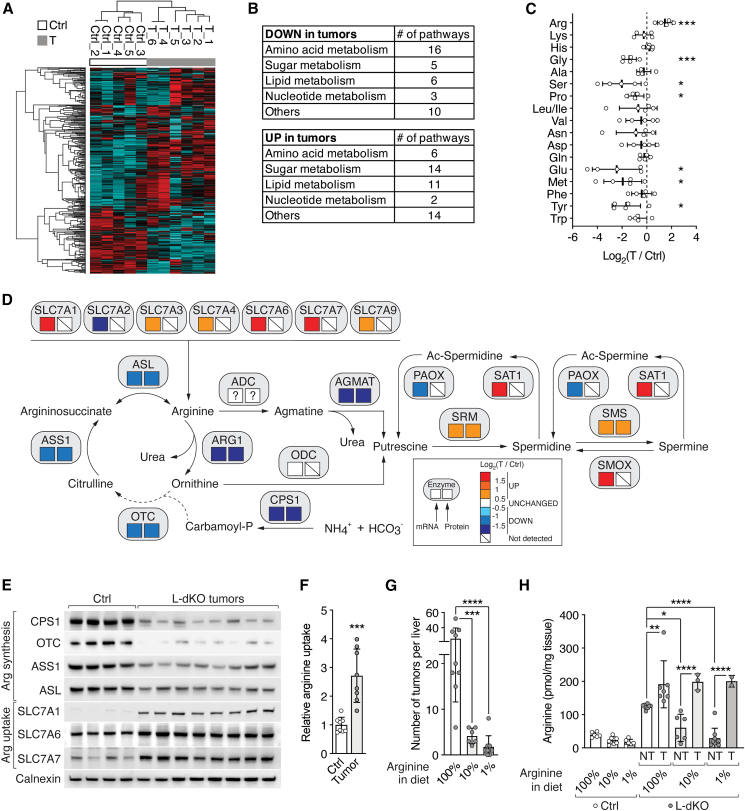
Arginine is elevated in liver tumors and promotes tumor formation (A) Hierarchical clustering of significantly altered metabolites from control (Ctrl) liver and tumor tissues (T) from liver-specific *Tsc1* and *Pten* double-knockout (hereafter, L-dKO) mice. n = 5 (Ctrl), n = 6 (L-dKO). (B) Up- and downregulated metabolic pathways in L-dKO tumors compared to Ctrl liver tissues, summarized from MPWEA (see Table S1). (C) Amino acid profile of L-dKO tumor relative to Ctrl liver tissues (log_2_ ratio). n = 5. (D) Schematic representation of arginine and polyamine metabolism. Boxes below enzymes indicate changes in mRNA (left box) and protein (right box) levels in L-dKO tumors compared to Ctrl livers, respectively. Color coding according to level of log_2_-fold change as indicated. SMOX, spermine oxidase; SAT1, spermidine/spermine N-acetyltransferase 1; PAOX, polyamine oxidase; “?” indicates unknown identity. n = 6 (Ctrl), n = 12 (L-dKO). (E) Immunoblots of arginine-synthesizing enzymes (CPS1, OTC, ASS1, and ASL) and arginine transporters (SLC7A1, SLC7A6, and SLC7A7) in Ctrl liver and L-dKO tumor tissues. Calnexin serves as loading control. n = 4 (Ctrl), n = 8 (L-dKO). (F) Relative ^3^H-arginine uptake into Ctrl liver and L-dKO tumor tissues. n = 8. (G) Number of macroscopic tumors per liver of L-dKO mice fed diets containing standard content (100%), 10%, or 1% of arginine for 8–20 weeks of age. n = 6–9. (H) Arginine content in Ctrl liver and L-dKO non-tumor (NT) and tumor (T) tissues of mice fed with arginine-modified diets. n = 3–9. ^∗^p < 0.05, ^∗∗^p < 0.01, ^∗∗∗^p < 0.001, ^∗∗∗∗^p < 0.0001 by unpaired t test (C and F) and one-way ANOVA (G and H).

**Figure S1:**
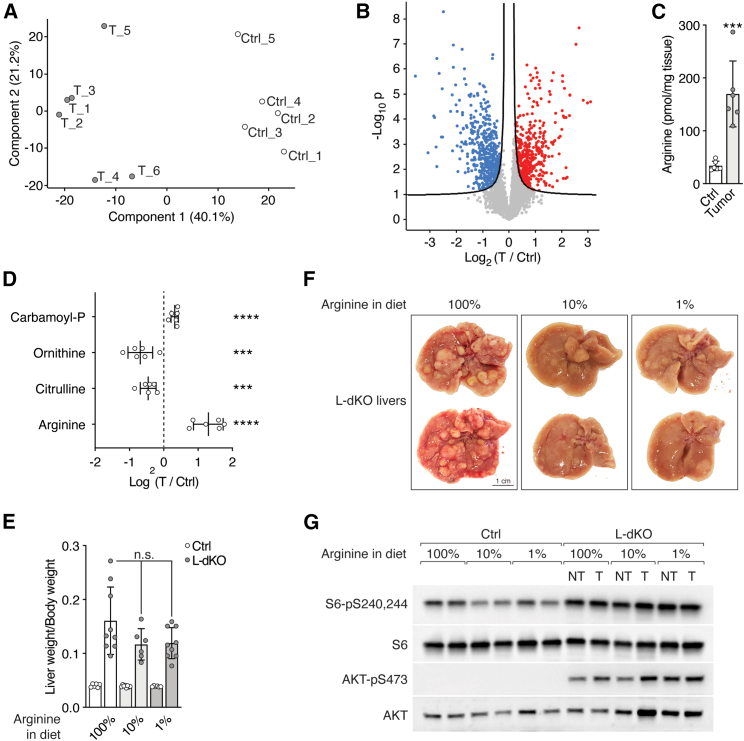
Arginine is increased in liver tumors, and high levels of arginine are required for tumorigenicity, related to Figure 1 (A) PCA analysis of untargeted metabolomics. n = 5 (Ctrl), n = 6 (T from L-dKO). (B) Volcano plot of the −log_10_(adjusted p value) against the log_2_ fold-change of metabolites detected in untargeted metabolomics. Blue and red dots indicate significantly decreased and increased metabolites in tumors (T) compared to Ctrl tissues, respectively. (C) Arginine content in Ctrl liver and L-dKO tumor tissues, as assessed by ELISA. n = 6. (D) Urea cycle metabolites in L-dKO tumors relative to Ctrl liver tissues (log_2_ ratio). n = 5 (Ctrl), n = 6 (L-dKO). (E) Liver-to-body-weight ratio of Ctrl and L-dKO mice fed with arginine-modified diets. n = 6–10. (F) Representative images of livers from L-dKO mice fed with arginine-modified diets. (G) Immunoblot analysis of mTOR signaling in Ctrl liver and L-dKO tumor tissues from mice fed with arginine-modified diets. Sustained high mTOR signaling in L-dKO livers underlies hepatomegaly. Total S6 and total AKT serve as loading controls. n = 2. n.s. = not significant; ^∗∗∗^p < 0.001, ^∗∗∗∗^p < 0.0001 by unpaired t test (C), multiple t test (D), and one-way ANOVA (E).

To identify metabolic alterations in HCC, we performed untargeted metabolomics^22^ on liver tumors isolated from a previously described mTOR-driven HCC mouse model.^23^^,^^24^^,^^25^ In this mouse model, constitutively high mTOR signaling due to liver-specific double knockout of the tumor suppressors TSC1 and PTEN (hereafter referred to as L-dKO) drives the sequential development of hepatomegaly, hepatosteatosis, steatohepatitis, and multiple high-grade HCC within 20 weeks of age.^23^^,^^24^ The metabolic profiles of tumors and control liver tissues were distinct, as revealed by principal component analysis (PCA) and hierarchical clustering (Figures 1A and S1A). 3,467 ions could be assigned to at least one known metabolite (see STAR Methods), of which 916 were significantly altered in abundance in L-dKO tumors (Figure S1B). Metabolic pathway enrichment analysis (MPWEA) indicated that amino acid metabolic pathways, and in particular arginine metabolism, were strongly altered in L-dKO tumors (Figure 1B; Table S1). To confirm effects on amino acid metabolism, we measured levels of individual amino acids by targeted liquid chromatography with tandem mass spectrometry (LC-MS/MS). Interestingly, arginine levels were elevated in L-dKO tumors, while the amounts of all other amino acids were either unchanged or decreased (Figures 1C, S1C, and S1D). This observation was surprising, as liver tumors are frequently deficient in arginine synthesis.^16^^,^^26^^,^^27^

Transcriptomic and proteomic analyses of L-dKO tumors^25^ also revealed broad deregulation of arginine metabolism (Figure 1D). Consistent with previous reports,^16^^,^^26^ the urea cycle, which produces arginine in the process of detoxifying excess ammonium, was transcriptionally downregulated in L-dKO tumors. Decreased expression of the urea cycle enzymes carbamoyl phosphate synthetase 1 (CPS1), ornithine transcarbamylase (OTC), ASS1, and argininosuccinate lyase (ASL) was confirmed by immunoblotting (Figure 1E). Furthermore, the urea cycle metabolites ornithine and citrulline were decreased in the liver tumors (Figure S1D). Suppression of the urea cycle makes cells dependent on extracellular arginine.^3^^,^^26^ Accordingly, several transporters of the solute carrier 7A family (SLC7A1, SLC7A3, SLC7A4, SLC7A6, SLC7A7, and SLC7A9), which mediate arginine uptake,^28^ were transcriptionally upregulated in L-dKO tumors (Figures 1D and 1E). An *ex vivo* arginine transport assay confirmed that arginine uptake is indeed increased in liver tumors (Figure 1F). Thus, L-dKO tumors increase arginine uptake to compensate for downregulation of arginine synthesis.

Next, we investigated if high levels of arginine are critical for liver tumor development. L-dKO and control mice were fed diets that contained 10% or 1% of normal levels of arginine found in the standard diet (100% arginine) from 8 to 20 weeks of age. Control mice were not affected by the arginine-restricted diets, as assessed by liver-to-body weight ratio (Figure S1E). L-dKO mice displayed characteristic hepatomegaly even upon arginine restriction, again suggesting that the decreased arginine in the diets was not limiting for growth (Figures S1E–S1G). However, the arginine-restricted diets significantly reduced tumor burden (Figures 1G and S1F). Thus, high levels of cellular arginine are critical for the development of liver tumors.

We note that arginine levels were also higher in presumably non-tumor liver tissue of L-dKO mice compared to normal liver tissue of control mice in normal diet conditions. This is likely due to the fact that at 20 weeks of age, L-dKO mice display multiple macroscopically visible tumors and numerous microscopic tumors in an overall damaged liver (Figures 1G and S1F), precluding the isolation of “clean” non-tumor tissue. However, arginine levels were significantly lower in non-tumor liver tissue of L-dKO mice upon dietary arginine restriction, but unaffected (i.e., still elevated) in all tumors (Figure 1H). This strict correlation of high arginine levels in tumors again suggests that high levels of cellular arginine are critical for tumorigenicity.

### Loss of ARG1 and AGMAT preserves oncogenic arginine levels

**Figure 2:**
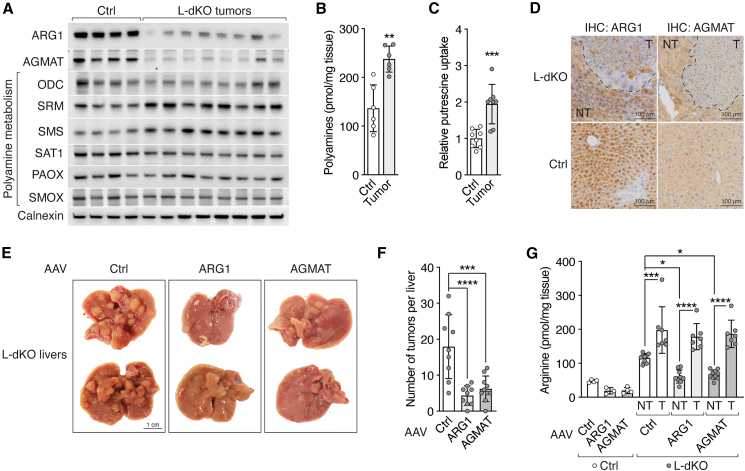
Loss of ARG1 and AGMAT enhances liver tumor formation (A) Immunoblots of arginine-to-polyamine-converting enzymes (ARG1 and AGMAT) and polyamine metabolism enzymes (ODC, SRM, SMS, SAT1, PAOX, and SMOX) in Ctrl liver and L-dKO tumor tissues. Calnexin serves as loading control (same samples were used as in Figure 1E). n = 4 (Ctrl), n = 8 (L-dKO). (B) Total polyamine content in Ctrl liver and L-dKO tumor tissues. n = 6. (C) Relative ^3^H-putrescine uptake into Ctrl liver and L-dKO tumor tissues. n = 8. (D) Immunohistochemistry of Ctrl and L-dKO liver tissues stained for ARG1 or AGMAT. NT, adjacent non-tumor tissue; T, tumor. (E) Representative images of livers from L-dKO mice injected with AAV-Ctrl, AAV-ARG1, or AAV-AGMAT. (F) Number of macroscopic tumors per liver of L-dKO mice injected with AAV-Ctrl, AAV-ARG1, or AAV-AGMAT. n = 9–10. (G) Arginine content in Ctrl liver and L-dKO non-tumor (NT) and tumor (T) tissues of mice injected with AAV-Ctrl, AAV-ARG1, or AAV-AGMAT. n = 4–10. ^∗^p < 0.05, ^∗∗^p < 0.01. ^∗∗∗^p < 0.001, ^∗∗∗∗^p < 0.0001 by unpaired t test (B and C) and one-way ANOVA (F and G).

Arginine is a precursor for polyamines, which are present in high concentration (up to millimolar) in cells.^29^ Thus, conversion of arginine to polyamines consumes a large amount of arginine. Polyamines, of which the major species are putrescine, spermidine, and spermine, are essential for cell growth and present in elevated levels in various cancers.^29^ Expression of polyamine metabolism enzymes was altered in tumors of L-dKO mice (Figure 1D). Importantly, arginase 1 (ARG1) and agmatinase (AGMAT), which catalyze arginine-to-polyamine conversion via two parallel pathways, were transcriptionally downregulated in L-dKO tumors (Figures 1D and 2A). ARG1 cleaves arginine to produce urea and ornithine in the last step of the urea cycle. Ornithine decarboxylase (ODC) then decarboxylates ornithine to produce putrescine. In the parallel, less-understood pathway, an unknown enzyme decarboxylates arginine to produce agmatine, which is then converted to putrescine by AGMAT. Spermidine synthase (SRM) and spermine synthase (SMS), which sequentially produce spermidine and spermine from putrescine, were transcriptionally upregulated in L-dKO tumors (Figures 1D and 2A). Protein levels of other polyamine metabolism enzymes were unchanged (Figure 2A).

**Figure S2:**
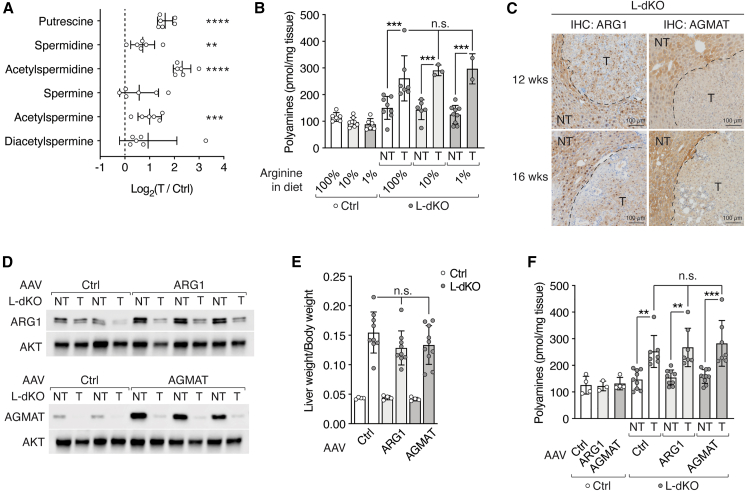
Loss of ARG1 and AGMAT promote tumorgenicity by sustaining high levels of arginine, related to Figure 2 (A) Polyamine species in L-dKO tumors relative to Ctrl liver tissues (log_2_ ratio). n = 5 (Ctrl), n = 6 (L-dKO). (B) Total polyamine content in Ctrl liver and L-dKO non-tumor (NT) and tumor (T) tissues of mice fed with arginine-modified diets. n = 3–9. (C) Immunohistochemistry of Ctrl and L-dKO liver tissues from 12- and 16-week-old mice stained for ARG1 or AGMAT proteins, respectively. NT, adjacent non-tumor tissue; T, tumor. (D) Immunoblots of ARG1 and AGMAT in paired L-dKO non-tumor (NT) and tumor (T) tissues from mice injected with AAV-Ctrl, AAV-ARG1, or AAV-AGMAT. AKT serves as loading control. n = 2 (AAV-Ctrl), n = 3 (AAV-ARG1), and n = 3 (AAV-AGMAT). (E) Liver-to-body-weight ratio of Ctrl and L-dKO mice injected with AAV-Ctrl, AAV-ARG1, or AAV-AGMAT. n = 4–10. (F) Total polyamine content in Ctrl liver and L-dKO non-tumor (NT) and tumor (T) tissues of mice injected with AAV-Ctrl, AAV-ARG1, or AAV-AGMAT. n = 4–10. n.s. = not significant; ^∗∗^p < 0.01, ^∗∗∗^p < 0.001, ^∗∗∗∗^p < 0.0001 by multiple t test (A) and one-way ANOVA (B, E, and F).

How do the observed changes in polyamine biosynthesis enzymes affect polyamine levels in L-dKO tumors? Consistent with reports on other cancers,^30^^,^^31^^,^^32^^,^^33^ total polyamine levels were increased in L-dKO tumors, as measured by a fluorometric assay (Figure 2B) and our untargeted metabolomics (Figure S2A). The reason for increased polyamines in cancer remains to be determined.

Given that ARG1 and AGMAT are downregulated in L-dKO tumors (Figures 1D and 2A), how do the tumors accumulate polyamines? An arginine-restricted diet did not decrease polyamine levels in L-dKO tumors (Figure S2B), suggesting that the accumulated polyamines are not derived from endogenous pools of arginine but rather from increased polyamine uptake. Indeed, putrescine uptake was increased in liver tumors (Figure 2C). Thus, the intracellular pools of polyamines and arginine are uncoupled, indicating that tumors do not accumulate arginine to produce polyamines.

Why are ARG1 and AGMAT downregulated in tumor cells? We speculated that loss of ARG1 and AGMAT, i.e., reduced arginine consumption, preserves the high levels of arginine that we found are critical for liver tumor development. Investigating this hypothesis, we first confirmed that loss of ARG1 and AGMAT expression is confined to tumors by immunohistochemistry (IHC) (Figure 2D). Next, to determine if expression of ARG1 and AGMAT declines early in tumor development, we performed IHC on livers of 12- and 16-week-old L-dKO mice. We note that 12 weeks is the earliest time point at which defined tumors can be detected in L-dKO livers. Interestingly, expression of both ARG1 and AGMAT was already decreased in tumors of 12- and 16-week-old L-dKO mice (Figure S2C). Thus, downregulation of ARG1 and AGMAT appears to be an early, critical event in liver tumorigenesis, possibly to preserve high levels of arginine. To test this, we injected 8-week-old L-dKO and control mice with a hepatocyte-specific adeno-associated virus (AAV)^34^ expressing ARG1 (AAV-ARG1) or AGMAT (AAV-AGMAT) (Figure S2D). Similar to our dietary arginine restriction experiments, all AAV-injected L-dKO mice developed hepatomegaly (Figures 2E and S2E), but L-dKO mice injected with AAV-ARG1 or AAV-AGMAT developed significantly fewer tumors per liver (Figures 2E and 2F) compared to mice injected with control virus. We also observed that overexpression of ARG1 or AGMAT after AAV injection was detected only in non-tumor liver tissue of L-dKO mice, whereas the few “escaper” tumors that appeared expressed low levels of ARG1 and AGMAT (Figure S2D). Importantly, AAV-ARG1 or AAV-AGMAT injection decreased arginine levels in non-tumor tissue but not in the few escaper tumors (Figure 2G), again indicating a strong correlation between high arginine levels and tumorigenicity. Neither AAV-ARG1 nor AAV-AGMAT had an effect on polyamine levels in normal liver tissue of control mice, in non-tumor tissue of L-dKO mice, or on the elevated polyamine levels in L-dKO tumors (Figure S2F). Taken together, our results suggest that ARG1 and AGMAT are suppressed to reduce arginine consumption and thereby preserve high levels of unmetabolized arginine required for tumorigenesis. In practical terms, low ARG1 and AGMAT expression can be viewed as a high arginine condition.

### Arginine determines expression of metabolic genes

**Figure S3:**
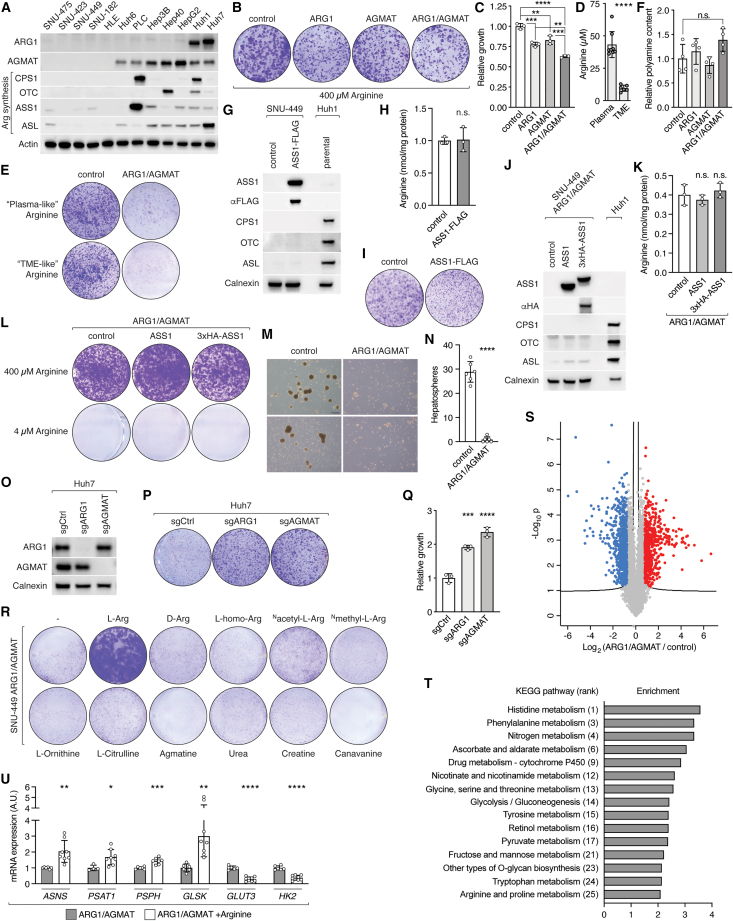
ARG1 and AGMAT expression determine metabolism and growth of liver cancer cells, related to Figure 3 (A) Immunoblots of ARG1, AGMAT, CPS1, OTC, ASS1, and ASL expression in human liver cancer cell lines. Actin serves as loading control. (B) Representative clonogenic growth assay of control, ARG1-, and/or AGMAT-expressing SNU-449 cells grown in standard, arginine-rich DMEM (i.e., 400 μM) medium. (C) Relative clonogenic growth of control, ARG1-, and/or AGMAT- expressing SNU-449 cells grown in standard, arginine-rich DMEM medium. N = 3. (D) Arginine content in plasma and TME of L-dKO mice. n = 8 (plasma), n = 6 (TME). (E) Representative clonogenic growth assay of control and ARG1/AGMAT-expressing SNU-449 cells grown in medium containing 100 μM arginine (“plasma-like”) or 20 μM arginine (“TME-like”). (F) Relative polyamine content of control, ARG1-, and/or AGMAT-expressing SNU-449 cells. N = 4. (G) Immunoblots of SNU-449 cells upon stable overexpression of ASS1-FLAG. Huh1 cells serve as control for expression of arginine synthesis enzymes. Calnexin serves as loading control. (H) Arginine content of control or ASS1-FLAG-overexpressing SNU-449 cells. (I) Representative clonogenic growth assay of control or ASS1-FLAG-overexpressing SNU-449 cells grown under arginine-restricted conditions. (J) Immunoblots of ARG1/AGMAT-expressing SNU-449 cells upon stable overexpression of ASS1 or 3xHA-ASS1. Huh1 cells serve as control for expression of arginine synthesis enzymes. Calnexin serves as loading control. (K) Arginine content of control, ASS1-, or 3xHA-ASS1-overexpressing SNU-449 ARG1/AGMAT cells. (L) Clonogenic growth assay of control, ASS1-, or 3xHA-ASS1-overexpressing SNU-449 ARG1/AGMAT cells grown under arginine-rich (400 μM) or arginine-restricted (4 μM) conditions. (M) Representative images of hepatospheres of control and ARG1/AGMAT-expressing SNU-449 cells grown in arginine-restricted medium in ultra-low attachment plates. Scale bar, 100 μm. (N) Number of hepatospheres (as in G). N = 6. (O) Immunoblot analyses of ARG1 and AGMAT in sgCtrl, sgARG1, and sgAGMAT Huh7 cells. Calnexin serves as loading control. (P) Representative clonogenic growth assay of sgCtrl, sgARG1, and sgAGMAT Huh7 cells. (Q) Relative clonogenic growth of sgCtrl, sgARG1, and sgAGMAT Huh7 cells. N = 3. (R) Clonogenic growth of ARG1/AGMAT-expressing SNU-449 cells grown in arginine-restricted medium in the presence of 400 μM of indicated metabolites. (S) Volcano plot of the −log_10_(adjusted p value) against the log_2_ fold-change of the differentially expressed genes in ARG1/AGMAT-expressing compared to control SNU-449 cells. Blue and red dots indicate significantly decreased and increased gene expression, respectively. (T) Deregulated metabolic pathways (within top 25 of all deregulated pathways; see Table S2) in ARG1/AGMAT-expressing compared to control SNU-449 cells after PWEA (using KEGG pathways, presented by enrichment factor) of differentially expressed genes from RNA-seq. (U) mRNA levels of *ASNS*, *PSAT1*, *PSPH*, *GLSK*, *GLUT3*, and *HK2* in ARG1/AGMAT-expressing SNU-449 cells grown in arginine-restricted medium with or without supplementation of excess arginine (i.e., 4 mM equal to 10× compared to standard DMEM medium) for 16 h. N = 4–8. n.s. = not significant; ^∗^p < 0.05, ^∗∗^p < 0.01, ^∗∗∗^p < 0.001, ^∗∗∗∗^p < 0.0001 by one-way ANOVA (C, F, K, and Q) and unpaired t test (D, H, N, and U).

**Figure 3:**
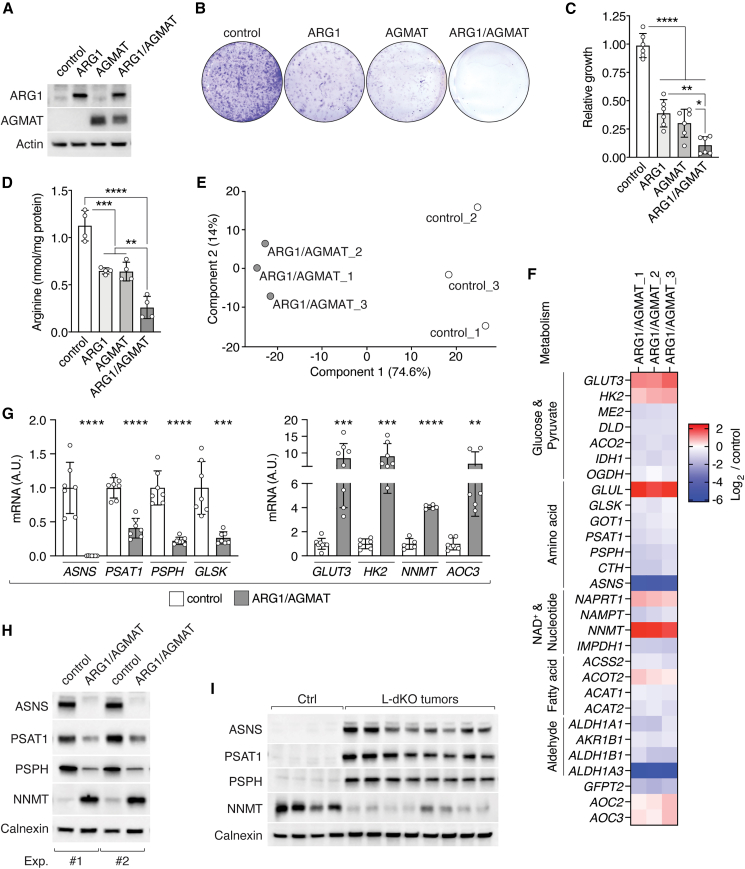
ARG1/AGMAT determine metabolic gene expression via arginine (A) Immunoblots of SNU-449 cells upon stable expression of ARG1 and/or AGMAT. Actin serves as loading control. (B) Representative clonogenic growth assay of control, ARG1-, and/or AGMAT-expressing SNU-449 cells grown in arginine-restricted medium. (C) Relative clonogenic growth of control, ARG1-, and/or AGMAT- expressing SNU-449 cells. N = 6. (D) Arginine content of control, ARG1-, and/or AGMAT-expressing SNU-449 cells. N = 4. (E) PCA analysis of RNA-seq data of control and ARG1/AGMAT-expressing SNU-449 cells. (F) Heatmap of a subset of differentially expressed metabolic genes in ARG1/AGMAT-expressing compared to control SNU-449 cells (log_2_ fold-change). (G) mRNA levels of *ASNS*, *PSAT1*, *PSPH*, *GLSK*, *GLUT3*, *HK2*, *NNMT,* and *AOC3* in control and ARG1/AGMAT-expressing SNU-449 cells. N = 5–7. (H) Immunoblots of ASNS, PSAT, PSPH, and NNMT from two independent experiments of control and ARG1/AGMAT-expressing SNU-449 cells. Calnexin serves as loading control. (I) Immunoblots of ASNS, PSAT, PSPH, and NNMT of Ctrl liver and L-dKO tumor tissues. Calnexin serves as loading control. n = 4 (Ctrl), n = 8 (L-dKO). ^∗^p < 0.05, ^∗∗^p < 0.01, ^∗∗∗^p < 0.001, ^∗∗∗∗^p < 0.0001 by one-way ANOVA (C and D) and unpaired t test (G).

To further investigate the role of unmetabolized arginine in HCC, we first screened a panel of human liver cancer cell lines for loss of ARG1, AGMAT, and arginine synthesis enzymes to find an *in vitro* experimental system that phenocopies L-dKO tumors, selecting the SNU-449 cell line (Figure S3A). To confirm the utility of SNU-449 cells as a proxy for L-dKO tumors, we stably expressed ARG1, AGMAT, or both ARG1 and AGMAT (hereafter ARG1/AGMAT) in these cells using a lentivirus system (Figure 3A). Expression of ARG1 and/or AGMAT mildly reduced clonogenic growth of SNU-449 cells in standard Dulbecco's Modified Eagle Medium (DMEM) cell culture medium, which contains a supraphysiological concentration of arginine (Figures S3B and S3C). In medium containing arginine at a physiological concentration resembling plasma^35^ or the tumor microenvironment (TME)^36^ (Figure S3D), ARG1 or AGMAT expression markedly reduced clonogenic growth of SNU-449 cells, while ARG1/AGMAT co-expression arrested growth (Figures 3B, 3C, and S3E). Furthermore, consistent with our *in vivo* experiments (arginine-restricted diets and AAV-mediated sustained ARG1 or AGMAT expression), SNU-449 cells expressing ARG1 or AGMAT displayed reduced arginine levels, and co-expression of ARG1/AGMAT further reduced arginine levels (Figure 3D). Expression of ARG1 and/or AGMAT did not increase total polyamine levels (Figure S3F), again consistent with our *in vivo* experiments. In summary, SNU-449 cells faithfully phenocopy L-dKO tumors and can thus be used as an *in vitro* proxy to study the oncogenic effect of arginine.

We note that ARG1 and AGMAT control arginine levels independent of ASS1, the rate-limiting enzyme in arginine synthesis. First, overexpression of ASS1 in SNU-449 parental or ARG1/AGMAT-expressing cells did not increase arginine levels or impact clonogenic growth, most likely because expression of three other arginine-synthesizing enzymes is suppressed in SNU-449 cells (Figures S3A and S3G–S3K). Second, ARG1/AGMAT expression reduced hepatosphere formation in SNU-449 cells (Figures S3M and S3N), and knockout of ARG1 or AGMAT increased clonogenic growth of Huh7 cells (Figures S3O–S3Q). Expression of ASS1 is elevated in hepatospheres^37^ and Huh7 cells (Figure S3A).

To further investigate the hypothesis that unmetabolized arginine promotes growth of liver cancer cells, we cultured ARG1/AGMAT-expressing SNU-449 cells in the presence or absence of high levels of arginine or several arginine-related metabolites. Only L-arginine, the physiologically relevant form of arginine, and no other related metabolite (D-arginine; canavanine; homo-, acetyl-, or methyl-arginine; or metabolites up- or downstream of arginine, such as citrulline, ornithine, agmatine, urea, and creatine), restored growth of ARG1/AGMAT-expressing SNU-449 cells (Figure S3R). Thus, specifically unmetabolized arginine promotes growth of cancer cells.

How does unmetabolized arginine promote growth of liver cancer cells? It has been reported that arginine impacts metabolism in immune and cancer cells. In T cells, arginine enhances oxidative phosphorylation (OXHPOS) and nucleotide synthesis.^18^ In leiomyosarcoma and melanoma cell lines, arginine starvation decreases glycolysis and enhances OXPHOS and serine synthesis.^21^ Conversely, in ASS1-negative breast cancer cell lines, arginine deprivation reduces OXPHOS, which leads to mitochondrial dysfunction.^20^ Interestingly, in prostate cancer cells, arginine promotes expression of OXPHOS genes via epigenetic regulation.^19^ Thus, we examined whether elevated arginine promotes metabolic reprogramming of liver cancer cells by regulating metabolic gene expression. To identify genes differentially expressed in response to arginine, we performed RNA sequencing (RNA-seq) on SNU-449 cells expressing ARG1/AGMAT and on SNU-449 control cells lacking ARG1/AGMAT. Based on the RNA-seq, ARG1/AGMAT-expressing cells separated from control cells in PCA (Figure 3E). 1,457 transcripts were differentially expressed in ARG1/AGMAT versus control cells (Figure S3S). PWEA (using KEGG pathways) revealed high frequency of terms related to metabolism (Table S2; Figure S3T). In line with reported effects of arginine on glycolysis in cancers,^19^^,^^20^^,^^21^ we observed increased expression of glucose transporter 3 (*GLUT3*) and hexokinase 2 (*HK2*) in ARG1/AGMAT-expressing cells (Figures 3F and 3G). However, ARG1/AGMAT-controlled arginine levels impacted liver cancer cell metabolism beyond central energy metabolism (Table S2; Figure S3T). Expression of ARG1/AGMAT also altered expression of genes in amino acid, NAD^+^, nucleotide, fatty acid, and aldehyde metabolism, among others (Figure 3F). From these altered metabolic pathways, we defined a gene signature, which we used as a readout in further experiments. This gene signature includes asparagine synthetase (*ASNS*), the serine biosynthesis genes phosphoserine aminotransferase 1 (*PSAT1*) and phosphoserine phosphatase (*PSPH*), glutaminase kidney isoform (*GLSK*, also known as *GLS1*), the glycolysis genes *GLUT3* and *HK2,* the NAD^+^ metabolic gene nicotinamide N-methyltransferase (*NNMT*), and the primary amine oxidase 3 (*AOC3*). *ASNS*, *PSAT1*, *PSPH*, and *GLSK* expression was decreased, while *GLUT3*, *HK2*, *NNMT,* and *AOC3* expression was increased in ARG1/AGMAT-expressing, i.e., low arginine, cells (Figures 3F and 3G). Accordingly, ASNS, PSAT1, and PSPH protein levels were decreased while NNMT protein levels were increased upon ARG1/AGMAT expression (Figure 3H). As expected, addition of excess arginine reversed the effect of ARG1/AGMT on expression of the signature genes (Figure S3U). Interestingly, in L-dKO liver tumors, in which ARG1 and AGMAT are suppressed (Figures 1D and 2A), ASNS, PSAT1, and PSPH protein levels are increased, while NNMT levels are decreased (Figure 3I). This correlation between ARG1/AGMAT status and expression of the signature genes further supports the hypothesis that ARG1/AGMAT-controlled arginine levels determine cancer metabolism. In summary, arginine controls oncogenic metabolism at the transcriptional level.

### Arginine-dependent ASNS expression further enhances arginine uptake

**Figure S4:**
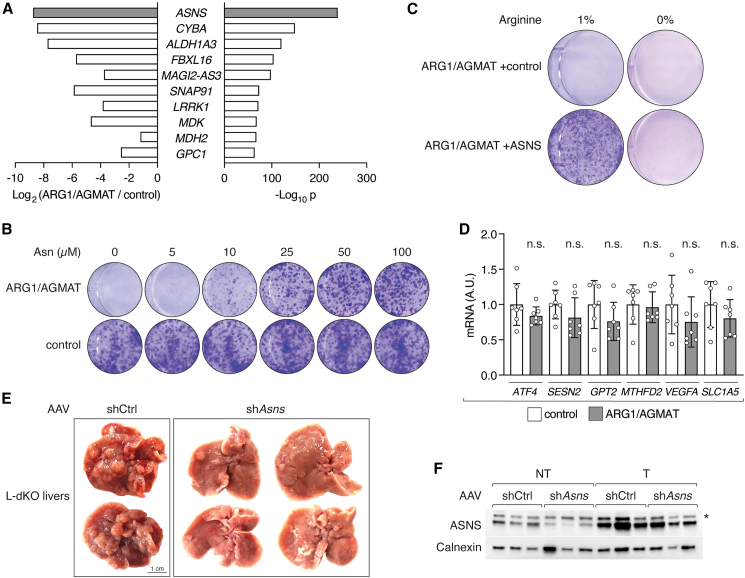
ARG1/AGMAT-regulated ASNS enhances arginine uptake required for tumorigenicity, related to Figure 4 (A) Top ten differentially expressed genes in ARG1/AGMAT-expressing compared to control SNU-449 cells by log_2_ fold-change (left) and −log_10_(adjusted p value) (right). (B) Clonogenic growth of control and ARG1/AGMAT-expressing SNU-449 cells grown in arginine-restricted medium supplemented with asparagine as indicated. (C) Clonogenic growth of ARG1/AGMAT+control or ARG1/AGMAT+ASNS-expressing SNU-449 cells grown in arginine-restricted or arginine-deficient medium. (D) mRNA levels of *ATF4* and ATF4 target genes *SESN2*, *GPT2*, *MTHFD2*, *VEGFA*, and *SLC1A5* in control and ARG1/AGMAT-expressing SNU-449 cells grown under arginine-restricted conditions. Unpaired t test; n.s. = not significant. N = 7. (E) Representative images of livers from L-dKO mice injected with AAV-shCtrl or AAV-sh*Asns*. (F) Immunoblot of ASNS in non-tumor (NT) and tumor (T) tissues of L-dKO mice injected with AAV-shCtrl or AAV-sh*Asns*. n = 3. Calnexin serves as loading control. ^∗^ indicates a cross-reaction.

**Figure 4:**
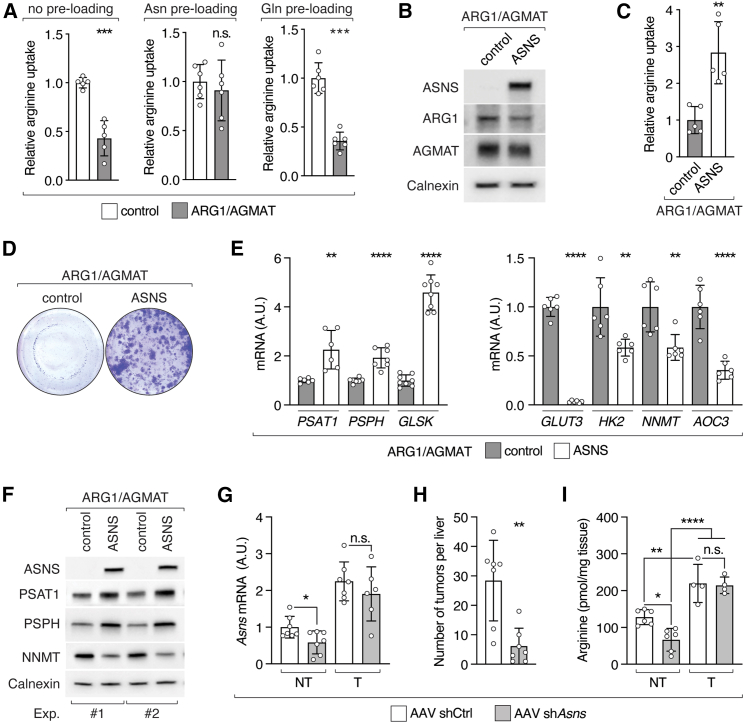
ASNS promotes arginine uptake in liver cancer (A) Relative ^3^H-arginine uptake in control and ARG1/AGMAT-expressing SNU-449 cells with or without pre-loading with asparagine (Asn) or glutamine (Gln). N = 5–6. (B) Immunoblots of ARG1/AGMAT-expressing SNU-449 cells upon stable expression of ASNS or control. Calnexin serves as loading control. (C) Relative ^3^H-arginine uptake in control and ASNS-expressing SNU-449 ARG1/AGMAT-expressing cells. N = 5. (D) Representative clonogenic growth assay of control and ASNS-expressing SNU-449 ARG1/AGMAT-expressing cells grown in arginine-restricted medium. (E) mRNA levels of *PSAT1*, *PSPH*, *GLSK*, *GLUT3*, *HK2*, *NNMT,* and *AOC3* in control and ASNS-expressing SNU-449 ARG1/AGMAT-expressing cells. N = 6–8. (F) Immunoblots of ASNS, PSAT, PSPH, and NNMT from two independent experiments of control and ASNS-expressing SNU-449 ARG1/AGMAT-expressing cells. Calnexin serves as loading control. (G) mRNA levels of *Asns* in L-dKO non-tumor (NT) and tumor (T) tissues of mice injected with AAV-shCtrl or AAV-sh*Asns*. n = 6–7. (H) Number of macroscopic tumors per liver in L-dKO mice injected with AAV-shCtrl or AAV-sh*Asns*. n = 7. (I) Arginine content in L-dKO non-tumor (NT) and tumor (T) tissues of mice injected with AAV-shCtrl or AAV-sh*Asns*. n = 4–6. n.s. = not significant; ^∗^p < 0.05, ^∗∗^p < 0.01, ^∗∗∗^p < 0.001, ^∗∗∗∗^p < 0.0001 by unpaired t test (A, C, E, G, and H) and one-way ANOVA (I).

Interestingly, ASNS was the most differentially expressed gene in ARG1/AGMAT-expressing cells compared to control cells (Figure S4A). Furthermore, asparagine has been suggested to serve as an anti-solute in cancer cells to facilitate uptake of essential amino acids, including arginine.^38^ Of note, we observed upregulation of uniporters and antiporters that mediate arginine uptake (Figures 1D and 1E). We therefore assessed uptake of arginine in ARG1/AGMAT-expressing SNU-449 cells in which ASNS is suppressed (Figures 3G and 3H). Indeed, arginine uptake was reduced in ARG1/AGMAT-expressing cells (Figure 4A). Arginine uptake could be restored by pre-loading cells with asparagine, but not with glutamine (Figure 4A).^39^ Furthermore, asparagine improved clonogenic growth of ARG1/AGMAT-expressing cells (Figure S4B). Thus, elevated arginine uptake in liver tumors appears to depend on ASNS-derived asparagine. To test this, we stably re-expressed ASNS in SNU-449 cells expressing ARG1/AGMAT (Figure 4B). Indeed, expression of ASNS was sufficient to increase arginine uptake and restore clonogenic growth of SNU-449 cells expressing ARG1/AGMAT in an arginine-dependent manner (Figures 4C, 4D, and S4C). Furthermore, increased arginine uptake also restored expression of the signature genes. *PSAT1*, *PSPH*, and *GLSK* expression was increased while *GLUT3*, *HK2*, *NNMT* and *AOC3* expression was decreased upon ASNS expression in ARG1/AGMAT-expressing cells (Figures 4E and 4F).

We note that *ASNS* expression is enhanced by the transcription factor ATF4 of the integrated stress response (ISR),^40^ which is commonly active in cancer.^41^ However, *ATF4* and ATF4 target genes, other than *ASNS*, were not differentially expressed upon ARG1/AGMAT expression (Figure S4D). This suggests that ISR is not sufficient for enhanced ASNS expression, i.e., additional arginine-dependent factors are required.

To assess the importance of high ASNS expression for tumorigenesis *in vivo*, we knocked down *Asns* in 8-week-old L-dKO mice using hepatocyte-specific AAVs (Figure 4G). Indeed, tumor burden was reduced in L-dKO mice upon *Asns* knockdown (Figures 4H and S4E). Consistent with our previous *in vivo* experiments, *Asns* knockdown was detected in non-tumor but not in tumor tissues (Figures 4G and S4F). Moreover, arginine levels were decreased in non-tumor tissues upon *Asns* knockdown but elevated in tumor tissues, again supporting the notion that ASNS is critical for tumor formation by promoting arginine uptake into liver cancer cells (Figure 4I). Altogether, we conclude that loss of ARG1 and AGMAT enhances arginine accumulation, leading to arginine-dependent expression of ASNS. ASNS-derived asparagine further enhances arginine uptake, creating a positive feedback loop. Furthermore, the above suggests that high levels of arginine promote tumorigenesis, at least in part, by metabolic reprogramming.

### Arginine specifically binds RBM39

**Figure S5:**
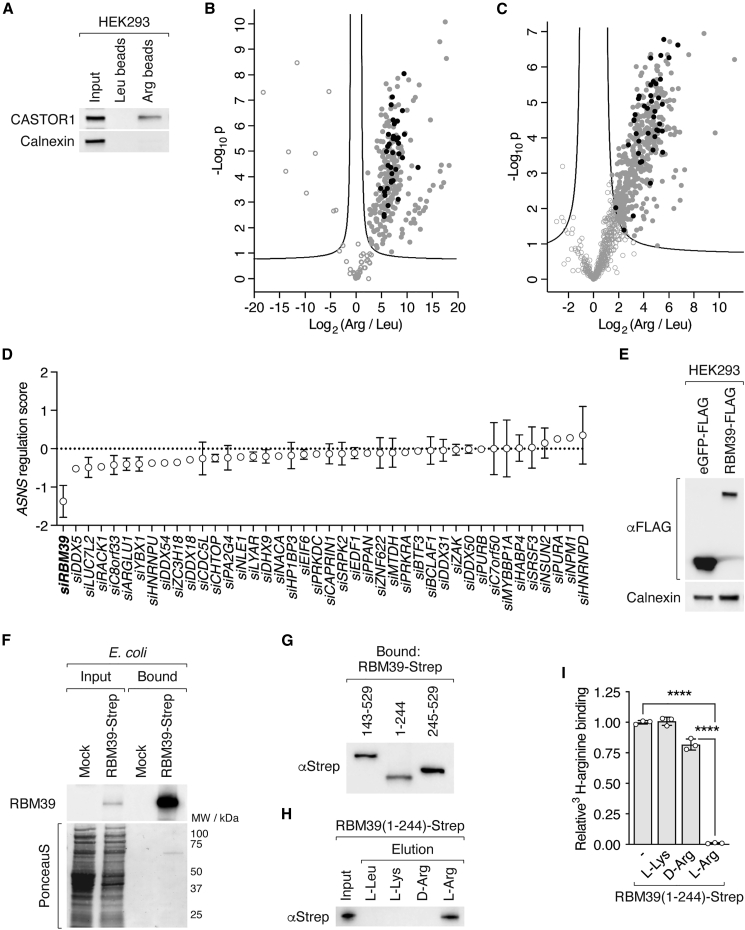
RBM39 is an arginine-binding protein, related to Figure 5 (A) Immunoblot of CASTOR1 in HEK293 cell lysate (Input) and elution after purification with leucine (Leu)- and arginine (Arg)-coupled agarose beads. Calnexin serves as input and negative control. (B) Volcano plot of the −log_10_(adjusted p value) against the log_2_ fold-change of 1,013 proteins identified by MS after purification from L-dKO tumor tissues by arginine (Arg)- compared to leucine (Leu)-coupled agarose beads. Black dots indicate 42 candidates that were selected for the siRNA screen (see D). (C) Volcano plot of the −log_10_(adjusted p value) against the log_2_ fold-change of 403 proteins identified by MS after purification from SNU-449 cells by arginine (Arg)- compared to leucine (Leu)-coupled agarose beads. Black dots indicate 42 candidates that were selected for the siRNA screen (see D). (D) siRNA screen of 42 candidates (see B and C). ASNS regulation score combines changes in mRNA levels of *ASNS* and knockdown efficiency of each candidate. N = 2. (E) Immunoblot of eGFP-FLAG and RBM39-FLAG expressed in HEK293 cells. Calnexin serves as loading control. (F) Immunoblot of RBM39 in mock and RBM39-Strep-expressing *E. coli* lysates (Input) and Strep-Tactin Sepharose immobilized fractions (Bound). PonceauS image indicates total protein in input. (G) Immunoblot of indicated RBM39-Strep fragments expressed in *E. coli* cells and immobilized with Strep-Tactin Sepharose (Bound). (H) Immunoblot of RBM39(1–244)-Strep expressed in *E. coli* cells (Input), purification with arginine (Arg)-coupled agarose beads and eluted with leucine (L-Leu), lysine (L-Lys), D-arginine (D-Arg), or arginine (L-Arg). (I) Relative ^3^H-arginine binding to RBM39(1–244)-Strep immunopurified from *E. coli* cells using Strep-Tactin Sepharose. ^3^H-arginine binding was competed with excess non-labeled lysine (L-Lys), D-arginine (D-Arg), or arginine (L-Arg) where indicated. One-way ANOVA, ^∗∗∗∗^p < 0.0001. N = 3.

**Figure 5:**
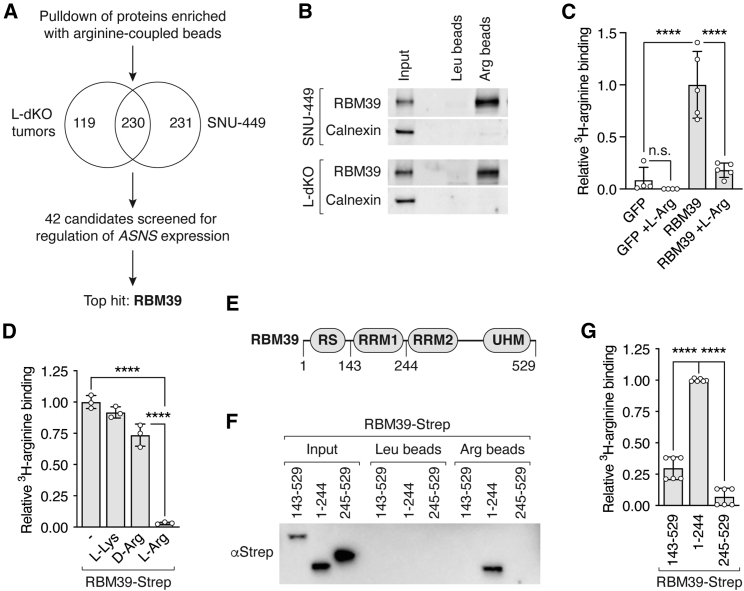
Arginine binds RBM39 (A) Schematic workflow that identified RBM39 as a potential arginine-binding transcriptional regulator of *ASNS*. (B) Immunoblots of RBM39 in L-dKO tumor tissue and SNU-449 cell lysate (Input) and elution after purification with leucine (Leu)- and arginine (Arg)-coupled agarose beads. Calnexin serves as input and negative control. (C) Relative ^3^H-arginine binding to GFP-FLAG or RBM39-FLAG immunopurified from HEK293 cells using anti-FLAG resin. ^3^H-arginine was competed with excess non-labeled arginine (L-Arg) where indicated. N = 4–5. (D) Relative ^3^H-arginine binding to RBM39-Strep immunopurified from *E. coli* cells using Strep-Tactin Sepharose. ^3^H-arginine was competed with excess non-labelled lysine (L-Lys), D-arginine (D-Arg), or arginine (L-Arg) where indicated. N = 3. (E) Schematic representation of RBM39. RS, arginine-serine-rich domain; RRM1 and RRM2, RNA recognition motif domains; UHM, U2AF homology motif domain. Numbers indicate amino acid positions. (F) Immunoblot of indicated RBM39-Strep fragments (Input). Elution after purification with leucine (Leu)- and arginine (Arg)-coupled agarose beads. (G) Relative ^3^H-arginine binding to indicated RBM39-Strep fragments immunopurified from *E. coli* cells using Strep-Tactin Sepharose. N = 6 (2 experiments with 3 technical replicates). n.s. = not significant, ^∗∗∗∗^p < 0.0001 by one-way ANOVA (C, D, and G).

How do high levels of arginine transcriptionally reprogram metabolism? A study in T cells suggested that arginine may interact with arginine-binding proteins to control metabolic gene expression.^18^ We hypothesized that such a mechanism may also exist in liver cancer cells. To identify potential arginine-binding proteins, we performed pull-down experiments with arginine-coupled beads, which we showed were able to purify the known arginine sensor CASTOR1^42^ (Figure S5A). We performed pull-down experiments with L-dKO tumor and SNU-449 cell lysates. Proteins were eluted with excess arginine and analyzed by mass spectrometry (MS) (Figure 5A). 349 and 461 proteins were significantly enriched in pull-downs from L-dKO tumor and SNU-449 lysates, respectively (Figures 5A, S5B, and S5C). 230 potential arginine-binding proteins were common to L-dKO tumors and SNU-449 cells (Figure 5A). Next, we knocked down the top 42 candidates associated with transcription, splicing, or signaling and assessed *ASNS* expression in SNU-449 cells. Knockdown of RBM39 strikingly reduced *ASNS* expression (Figure S5D). RBM39, also known as HCC1 and CAPERα, is an essential arginine-serine-rich RNA-binding protein involved in pre-mRNA splicing and transcription coactivation or co-repression.^43^ We confirmed by immunoblotting that RBM39 could be purified from L-dKO tumors and SNU-449 cells with arginine-coupled beads (Figure 5B). Furthermore, recombinant RBM39 immunopurified from mammalian or bacterial cells bound radiolabeled arginine in a manner that was effectively competed with excess “cold” L-arginine but not with lysine or D-arginine (Figures 5C, 5D, S5E, and S5F). Thus, arginine specifically binds RBM39.

RBM39 contains an N-terminal arginine-serine-rich (RS) domain, two RNA recognition motif domains (RRM1 and RRM2), and a C-terminal U2AF homology motif domain (UHM)^43^ (Figure 5E). To identify the arginine-binding region in RBM39, we expressed recombinant fragments of RBM39 in *E. coli*. Only RBM39(1–244), an N-terminal fragment containing the RS domain, bound arginine-coupled beads (Figure 5F) and radiolabeled arginine (Figures 5G and S5G). Again, binding was specific to L-arginine, as leucine, lysine, or D-arginine failed to elute RBM39(1–244) from arginine-coupled beads (Figure S5H) or compete with radiolabeled arginine for binding to RBM39(1–244) (Figure S5I). These findings suggest that arginine specifically binds the N-terminal region of RBM39, most likely the region N-terminal to the RRM1 domain, to promote at least *ASNS* expression.

### Arginine-bound RBM39 controls transcription of metabolic genes

**Figure S6:**
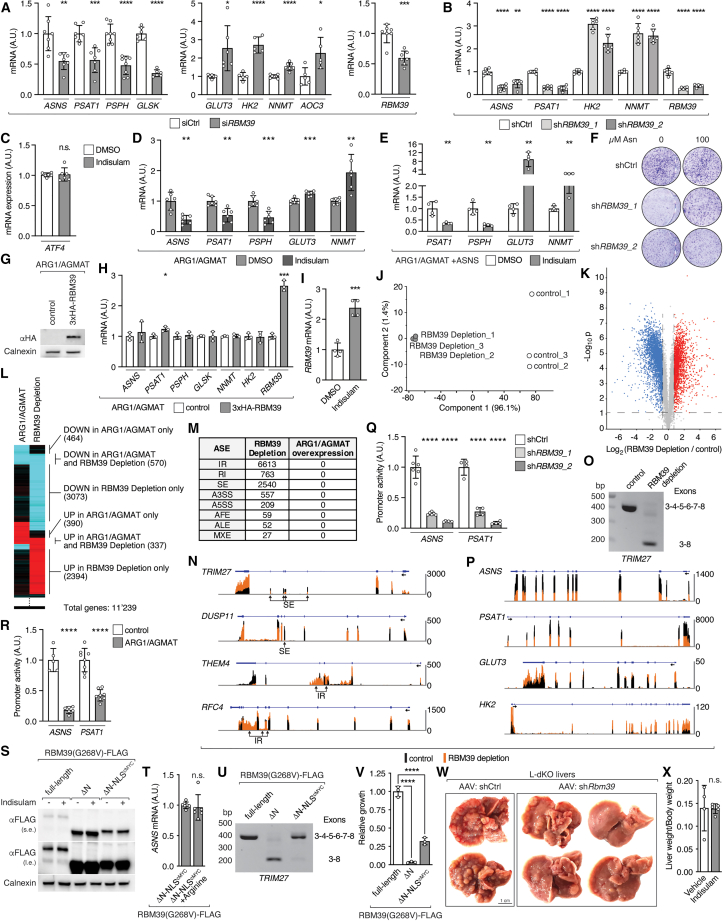
RBM39 requires arginine binding to transcriptionally control metabolic gene expression and tumorigenicity, related to Figure 6 (A) mRNA levels of *ASNS*, *PSAT1*, *PSPH*, *GLSK*, *GLUT3*, *HK2*, *NNMT*, *AOC3*, and *RBM39* upon si*RBM39* and siCtrl in SNU-449 cells. N = 5–7. (B) mRNA levels of *ASNS*, *PSAT1*, *HK2*, *NNMT*, and *RBM39* upon stable knockdown of *RBM39* (sh*RBM39_1* and sh*RBM39_2)* and shCtrl in SNU-449 cells. N = 5–6. (C) mRNA levels of *ATF4* in indisulam- or DMSO-treated SNU-449 cells. N = 6. (D) mRNA levels of *ASNS*, *PSAT1*, *PSPH*, *GLUT3*, and *NNMT* in indisulam- or DMSO-treated ARG1/AGMAT-expressing SNU-449 cells. N = 5–6. (E) mRNA levels of *PSAT1*, *PSPH*, *GLUT3*, and *NNMT* in indisulam- or DMSO-treated ARG1/AGMAT+ASNS-expressing SNU-449 cells. N = 4. (F) Representative clonogenic growth assay of SNU-449 shCtrl, sh*RBM39_1*, and sh*RBM39_2* cells grown under arginine-restricted conditions in the absence or presence of 100 μM asparagine. (G) Immunoblot of 3xHA-RBM39 expressed in ARG1/AGMAT-expressing SNU-449 cells. Calnexin serves as loading control. (H) mRNA levels of *ASNS*, *PSAT1*, *PSPH*, *GLSK*, *NNMT, HK2*, and *RBM39* in control and 3xHA-RBM39-expressing SNU-449 ARG1/AGMAT cells. N = 3. (I) mRNA levels of *RBM39* in indisulam- or DMSO-treated SNU-449 cells. N = 4. (J) PCA analysis of RNA-seq data of control and RBM39-depleted SNU-449 cells. (K) Volcano plot of the −log_10_(adjusted p value) against the log_2_ fold-change of differentially expressed genes in RBM39-depleted compared to control SNU-449 cells. Blue and red dots indicate significantly decreased and increased gene expression, respectively. (L) Clustering of the top 2,500 differentially expressed genes in ARG1/AGMAT-expressing compared to control SNU-449 cells with the differentially expressed genes in RBM39-depleted compared to control SNU-449 cells. Values of differentially expressed genes were binarized prior to clustering. (M) Table summarizing alternative splicing events (ASEs) detected in RNA-seq of control and RBM39-depleted SNU-449 cells and control and ARG1/AGMAT-expressing SNU-449 cells after analysis with the R package NxtIRFcore. IR, intron retention by algorithm; RI, intron retention curated; SE, skipped exon; A3SS, alternative 3′ splice site; A5SS, alternative 5′ splice site; AFE, alternative first exon; ALE, alternative last exon; MXE, mutually excluded exon (see also Table S4). (N) Read counts of *TRIM27* (Tripartite motif-containing protein 27), *DUSP11* (Dual specificity protein phosphatase 11), *THEM4* (Thioesterase superfamily member 4), and *RFC4* (Replication factor C subunit 4) from RNA-seq of control and RBM39-depleted SNU-449 cells displayed with integrated genome viewer (IGV). Regions highlighted with arrows indicate skipped exons (SE) or intron retention (IR). Blue line indicates introns, and blue boxes indicate exons. Arrow below blue line indicates gene orientation. (O) Representative endpoint PCR of *TRIM27* (exon 3–8) in control and RBM39-depleted cells (as in J). (P) Read counts of *ASNS*, *PSAT1*, *GLUT3*, and *HK2* from RNA-seq of control and RBM39-depleted SNU-449 cells displayed with IGV (as in N). (Q) Relative luciferase-based promoter activity of *ASNS* and *PSAT1* in SNU-449 shCtrl, sh*RBM39_1*, and sh*RBM39_2* cells grown under arginine-restricted conditions. N = 4–6. (R) Relative luciferase-based promoter activity of *ASNS* and *PSAT1* in control and ARG1/AGMAT-expressing SNU-449 cells grown under arginine-restricted conditions. N = 5–8. (S) Immunoblots of SNU-449 cells expressing full-length, ΔN, or ΔN-NLS^cMYC^ RBM39(G268V)-FLAG treated with indisulam or DMSO. Calnexin serves as loading control. s.e., short exposure; l.e., long exposure. (T) mRNA levels of *ASNS* in SNU-449 cells expressing ΔN-NLS^cMYC^ RBM39(G268V)-FLAG treated with indisulam for two days in arginine-restricted conditions or in arginine-repleted conditions (400 μM). N = 6. (U) Representative endpoint PCR of *TRIM27* (exon 3–8) in SNU-449 cells expressing full-length, ΔN, or ΔN-NLS^cMYC^ RBM39(G268V)-FLAG treated with indisulam. (V) Relative clonogenic growth of SNU-449 cells expressing full-length, ΔN, or ΔN-NLS^cMYC^ RBM39(G268V)-FLAG treated with indisulam. N = 3. (W) Representative images of livers from L-dKO mice injected with AAV-shCtrl or AAV-sh*Rbm39*. (X) Liver-to-body-weight ratio of L-dKO mice injected with indisulam or vehicle. n = 4 (vehicle), n = 5 (indisulam). n.s. = not significant; ^∗^p < 0.05, ^∗∗^p < 0.01, ^∗∗∗^p < 0.001, ^∗∗∗∗^p < 0.0001 by unpaired t test (A–E, H, I, Q, R, T, and X) and one-way ANOVA (V).

**Figure 6:**
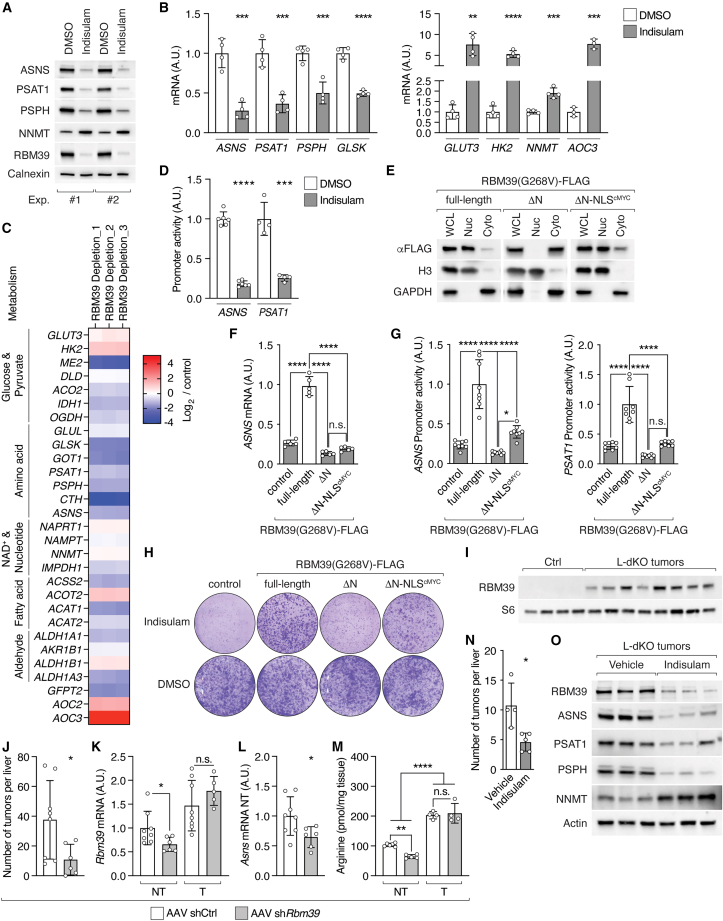
Arginine-bound RBM39 controls metabolic gene expression (A) Immunoblots of ASNS, PSAT, PSPH, NNMT, and RBM39 from two independent experiments of indisulam- or dimethyl sulfoxide (DMSO)-treated SNU-449 cells. Calnexin serves as loading control. (B) mRNA levels of *ASNS*, *PSAT1*, *PSPH*, *GLSK*, *GLUT3*, *HK2*, *NNMT,* and *AOC3* in indisulam- or DMSO-treated SNU-449 cells. N = 3–4. (C) Heatmap of a subset of differentially expressed metabolic genes in RBM39-depleted compared to control SNU-449 cells (log_2_ fold-change). (D) Relative luciferase-based promoter activity of *ASNS* and *PSAT1* in SNU-449 cells treated with indisulam or DMSO. N = 4 (*PSAT1*), N = 6 (*ASNS*). (E) Immunoblots upon fractionation of SNU-449 cells expressing full-length, ΔN, or ΔN-NLS^cMYC^ RBM39(G268V)-FLAG. WCL, whole cell lysate; Nuc, nuclear fraction; Cyto, cytoplasmic fraction. Histone3 (H3) and Glyceraldehyde-3-phosphate dehydrogenase (GAPDH) serve as fraction markers for Nuc and Cyto, respectively. (F) mRNA levels of *ASNS* in SNU-449 cells without (control) or with expression of full-length, ΔN, or ΔN-NLS^cMYC^ RBM39(G268V)-FLAG treated with indisulam for two days. N = 5. (G) Relative luciferase-based promoter activity of *ASNS* and *PSAT1* in SNU-449 cells without (control) or with expression of full-length, ΔN, or ΔN-NLS^cMYC^ RBM39(G268V)-FLAG treated with indisulam for two days. N = 8. (H) Representative clonogenic growth assay of SNU-449 cells without (control) or with expression of full-length, ΔN, or ΔN-NLS^cMYC^ RBM39(G268V)-FLAG grown in arginine-restricted medium and treated with DMSO or indisulam. (I) Immunoblot of RBM39 in Ctrl liver and L-dKO tumor tissues. Ribosomal protein S6 serves as loading control. n = 4 (Ctrl), n = 8 (L-dKO). (J) Number of macroscopic tumors per liver in mice injected with AAV-shCtrl or AAV-sh*Rbm39*. n = 8 (AAV-shCtrl), n = 6 (AAV-sh*Rbm39*). (K) mRNA levels of *Rbm39* in non-tumor (NT) and tumor (T) tissues of L-dKO mice injected with AAV-shCtrl or AAV-sh*Rbm39*. n = 5–8. (L) mRNA levels of *Asns* in non-tumor (NT) tissues of mice injected with AAV-shCtrl or AAV-sh*Rbm39*. n = 8 (AAV-shCtrl), n = 6 (AAV-sh*Rbm39*). (M) Arginine content in L-dKO non-tumor (NT) and tumor (T) tissues of mice injected with AAV-shCtrl or AAV-sh*Rbm39*. n = 4–6. (N) Number of macroscopic tumors per liver of L-dKO mice injected 7 times with 37.5 mg/kg indisulam or vehicle. n = 4 (vehicle), n = 5 (indisulam). (O) Immunoblots of RBM39, ASNS, PSAT1, PSPH, and NNMT from L-dKO tumor tissues (from mice as in N). Actin serves as loading control. n.s. = not significant; ^∗^p < 0.05, ^∗∗^p < 0.01, ^∗∗∗^p < 0.001, ^∗∗∗∗^p < 0.0001 by unpaired t test (B, D, J–L, and N) and one-way ANOVA (F, G, and M).

We next determined whether RBM39 affects expression of arginine-controlled signature genes (see “Arginine determines expression of metabolic genes”) other than *ASNS* in SNU-449 cells. Transient small interfering RNA (siRNA)-mediated or stable short hairpin RNA (shRNA)-mediated knockdown of *RBM39* decreased *PSAT1*, *PSPH*, and *GLSK* expression and increased *GLUT3, HK2, NNMT*, and *AOC3* expression (Figures S6A and S6B). We also depleted RBM39 with the aryl sulfonamide indisulam.^43^^,^^44^^,^^45^ Indisulam is a so-called “molecular glue” that converts RBM39 into a neo-substrate of the DCAF15-associated ubiquitin ligase complex, thereby inducing specific proteasomal degradation of RBM39.^44^^,^^45^ Treatment of SNU-449 cells with 10 μM indisulam reduced RBM39 protein levels (Figure 6A) and, more importantly, decreased *ASNS*, *PSAT1*, *PSPH*, and *GLSK* and increased *GLUT3*, *HK2*, *NNMT*, and *AOC3* but did not affect *ATF4* expression (Figures 6A, 6B, and S6C). Indisulam treatment also further aggravated the altered expression of signature genes in ARG1/AGMAT-expressing cells (Figure S6D) and blocked the effect of ASNS re-expression in ARG1/AGMAT-expressing cells (Figure S6E). Moreover, addition of excess asparagine (i.e., increasing arginine uptake) failed to restore clonogenic growth of SNU-449 cells with stable knockdown of *RBM39,* and overexpression of RBM39 failed to restore signature gene expression in ARG1/AGMAT-expressing SNU-449 cells (Figures S6F–S6H). These findings suggest that RBM39 controls metabolic gene expression in an arginine-dependent manner.

We next performed RNA-seq to determine how broadly RBM39 controls metabolic gene expression. In this case, we combined indisulam treatment and siRNA-mediated *RBM39* knockdown, since indisulam treatment increased *RBM39* mRNA levels (Figure S6I) and siRNA-mediated *RBM39* knockdown alone was incomplete (Figure S6A). RNA-seq samples from RBM39-depleted cells separated from control cells in PCA (Figure S6J). 7,113 transcripts were differentially expressed in RBM39-depleted cells (Figure S6K). To assess how many of these differentially expressed genes are potentially regulated by arginine through RBM39, we compared the top 2,500 genes deregulated upon arginine limitation, i.e., upon ARG1/AGMAT expression (see “Arginine determines expression of metabolic genes”), with the expression profile of RBM39-depleted cells (Figure S6L). Importantly, 907 of the 2,500 (36%) deregulated genes were similarly differentially expressed in RBM39-depleted cells. PWEA of these 907 genes revealed enrichment of metabolic pathways (Table S3). Strikingly, RBM39 depletion, like arginine depletion (by ARG1/AGMAT expression), altered expression of genes in glucose, pyruvate, amino acid, NAD^+^, nucleotide, fatty acid, and aldehyde metabolism (Figures 6C and 3F). Thus, arginine appears to control metabolic gene expression widely via RBM39.

RBM39 is a pre-mRNA splicing factor and transcription coactivator or co-repressor.^43^ We investigated whether RBM39 controls metabolic gene expression via pre-mRNA splicing or transcription by performing differential alternative splicing analysis on our RNA-seq datasets from SNU-449 cells (see “Arginine determines expression of metabolic genes” and “Arginine-bound RBM39 controls transcription of metabolic genes”). ARG/AGMAT expression (i.e., arginine restriction) did not impact splicing in SNU-449 cells (Figure S6M). In contrast, RBM39 depletion caused alternative splicing of many pre-mRNAs, including *TRIM27* pre-mRNA, as described previously^44^^,^^46^^,^^47^^,^^48^ (Figures S6M and S6O; Table S4). However, RBM39 depletion did not impact splicing of the metabolic signature genes (Figure S6P; Table S4), suggesting that RBM39 controls expression of metabolic genes via transcription rather than via splicing. To test this suggestion, we performed luciferase-based promoter activity assays using 1,000 bp fragments of the promoter regions of *ASNS* and *PSAT1*. RBM39 depletion by indisulam and stable *RBM39* knockdown reduced *ASNS* and *PSAT1* promoter activity (Figures 6D and S6Q). Importantly, ARG1/AGMAT expression also reduced *ASNS* and *PSAT1* promoter activity (Figure S6R), again suggesting that arginine and RBM39 control metabolic gene transcription.

To further investigate if RBM39 requires arginine binding to control transcription of metabolic genes, we expressed RBM39 wild-type and mutants in SNU-449 cells. In particular, we expressed recombinant, FLAG-tagged full-length RBM39; RBM39 lacking the N-terminal arginine-binding region (termed RBM39ΔN); or, since deletion of the N terminus of RBM39 prevents nuclear entry of RBM39,^49^ RBM39ΔN fused to the cMYC nuclear localization signal (termed RBM39ΔN-NLS^cMYC^) (Figure 6E). In all cases, the recombinant RBM39 versions contained a G268V mutation that confers resistance to indisulam (Figure S6S).^44^^,^^45^ The SNU-449 cells were treated with indisulam to deplete endogenous RBM39. Expression of *ASNS* was reduced in RBM39ΔN and RBM39ΔN-NLS^cMYC^ SNU-449 cells, but not in cells expressing full-length RBM39, even in the presence of high arginine (Figures 6F and S6T). Also, *ASNS* and *PSAT1* promoter activity was reduced in RBM39 arginine-binding-deficient mutants (Figure 6G). In contrast, splicing of *TRIM27* pre-mRNA was restored in RBM39ΔN-NLS^cMYC^ SNU-449 cells (Figure S6U). Thus, RBM39 requires its arginine-binding domain to control metabolic gene expression but not to mediate pre-mRNA splicing. Furthermore, clonogenic growth in the presence of indisulam was reduced in SNU-449 cells expressing arginine-binding-deficient RBM39 compared to full-length RBM39 (Figures 6H and S6V). We note that deletion of the N terminus can affect RBM39 function, and thus clonogenic growth, independently of arginine binding.

### RBM39 promotes tumorigenesis

RBM39 expression is elevated in L-dKO tumors compared to control liver tissues (Figure 6I). To investigate whether RBM39 is important for tumorigenesis, we knocked down *Rbm39* in 8-week-old L-dKO mice using hepatocyte-specific AAVs. *Rbm39* knockdown reduced tumor burden in L-dKO mice (Figures 6J and S6W), and as observed upon ARG1 or AGMAT overexpression and *Asns* knockdown, *Rbm39* knockdown was detected only in non-tumor tissue (Figure 6K). Moreover, knockdown of *Rbm39* correlated with decreased *Asns* mRNA and arginine levels in non-tumor tissue (Figures 6L and 6M), supporting the notion that RBM39 transcriptionally controls ASNS, which in turn promotes arginine uptake.

To confirm that RBM39 is essential for tumor development, we treated 16-week-old L-dKO mice with indisulam. Seven injections over two weeks were sufficient to reduce tumor progression without affecting liver-to-body weight ratio (Figures 6N and S6X). Importantly, indisulam treatment reduced RBM39 protein levels in L-dKO tumors and also reversed the arginine-induced effects of RBM39 on expression of ASNS and other metabolic enzymes (Figure 6O). Altogether, the above suggests that arginine-bound RBM39 transcriptionally reprograms metabolism and thereby promotes tumorigenicity.

### RBM39 is required for metabolic reprograming and tumor progression in HCC

**Figure 7:**
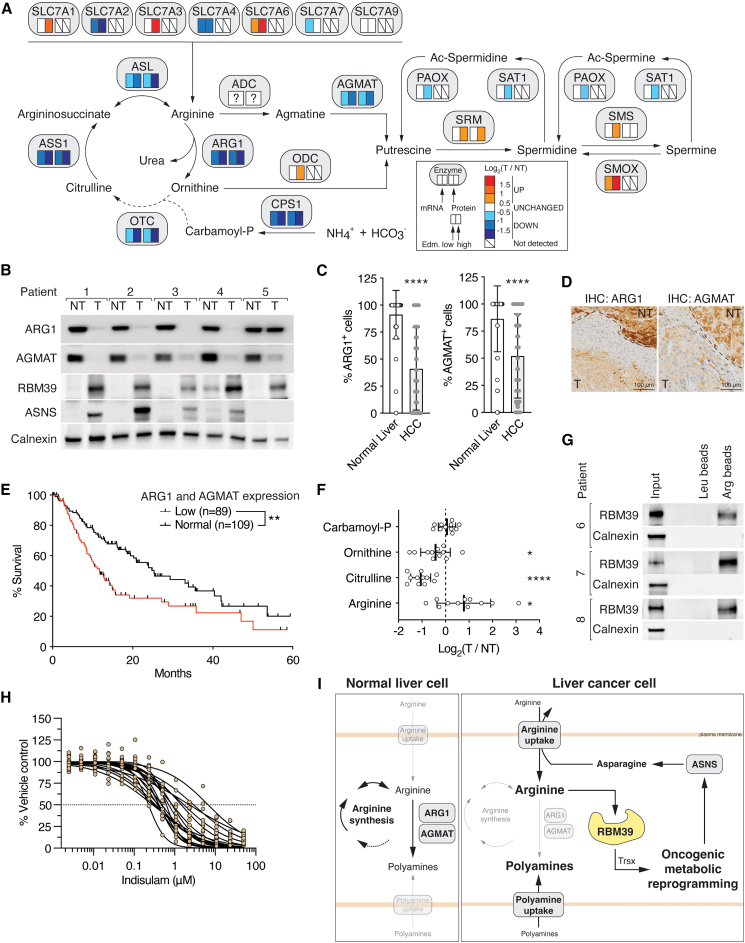
ARG1, AGMAT, arginine, and RBM39 in human HCC patients (A) Schematic representation of arginine and polyamine metabolism in HCC patients. Boxes below enzymes indicate changes in mRNA (left box) and protein (right box) levels in human HCC tumors (T) compared to paired non-tumor (NT) biopsies, respectively. Color coding according to level of log_2_ fold-change as indicated. “?” indicates unknown identity. Tumor aggressiveness is indicated by Edmondson-Steiner grade low (Edm. low, grade I and II) and high (Edm. high, grade III and IV). n = 73 (Edm. low) and n = 49 (Edm. high) for mRNA; n = 30 (Edm. low) and n = 21 (Edm. high) for protein. (B) Immunoblots of ARG1, AGMAT, RBM39, and ASNS in paired non-tumor (NT) and tumor (T) tissues of five HCC patients. Calnexin serves as loading control. (C) Tissue microarray for ARG1 and AGMAT. ARG1, normal liver n = 58, HCC n = 160; AGMAT, normal liver n = 49, HCC n = 142. (D) Representative IHC of ARG1 and AGMAT of an HCC patient (from C). Non-tumor, NT; tumor, T. (E) Kaplan-Meier survival estimate curve for The Cancer Genome Atlas Liver Hepatocellular Carcinoma (TCGA-LIHC) patients ranked by expression of *ARG1* and *AGMAT*. n = 89 (low), n = 109 (normal). (F) Urea cycle metabolites in tumors (T) relative to paired non-tumor (NT) liver tissues (log_2_ ratio). n = 11. (G) Immunoblots of RBM39 in tumor lysate (Input) and elution after purification with leucine (Leu)- or arginine (Arg)-coupled agarose beads from three HCC patients. Calnexin serves as input and negative control. (H) Dose-response curve of 20 HCC patient-derived organoids treated with indisulam. Data are presented as the percentage of control DMSO-treated tumor organoids. (I) Model. In liver cancer cells, loss of ARG1 and AGMAT preserves arginine, which in turn binds RBM39 to promote metabolic reprogramming. Arginine-RBM39-mediated ASNS expression further enhances arginine uptake. Trsx, transcription. ^∗^p < 0.05, ^∗∗^p < 0.01, ^∗∗∗∗^p < 0.0001 by unpaired t test (C), log rank test (E), and multiple t test (F).

**Figure S7:**
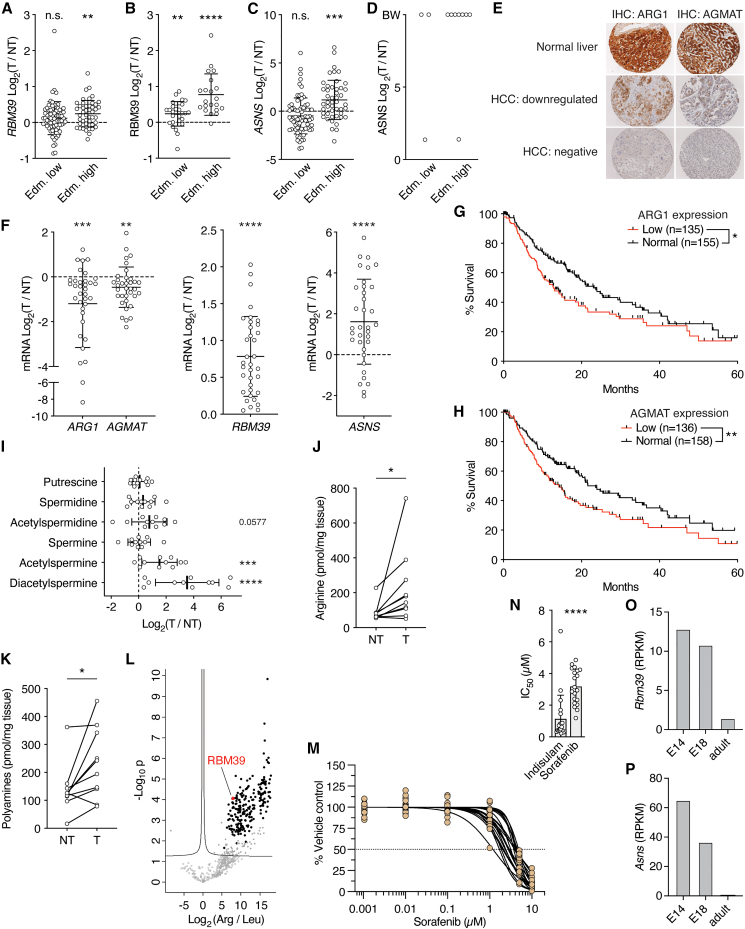
ARG1 and AGMAT are decreased and arginine, RBM39, and ASNS are increased in HCC patient tumors that are sensitive to RBM39 depletion by indisulam, related to Figure 7 (A) *RBM39* mRNA levels in liver tumor tissue (T) from HCC patients compared to adjacent non-tumor tissue (NT), displayed as log_2_ ratio. n = 73 (Edm. low), n = 49 (Edm. high). (B) RBM39 protein levels in liver tumor tissue (T) from HCC patients compared to adjacent non-tumor tissue (NT), displayed as log_2_ ratio. n = 30 (Edm. low), n = 21 (Edm. high). (C) *ASNS* mRNA levels in liver tumor tissue (T) from HCC patients compared to adjacent non-tumor tissue (NT), displayed as log_2_ ratio. n = 73 (Edm. low), n = 49 (Edm. high). (D) ASNS protein levels in liver tumor tissue (T) from HCC patients compared to adjacent non-tumor tissue (NT), displayed as log_2_ ratio, if applicable. BW, black-and-white, i.e., only detected in tumor tissues. n = 3 (Edm. low), n = 8 (Edm. high). (E) Staging of ARG1 and AGMAT IHC staining in tissue micro array. (F) mRNA expression of *ARG1*, *AGMAT*, *RBM39*, and *ASNS* in early-stage HCC (data from Jiang et al.^51^). log_2_ fold-change tumor (T) relative to non-tumor (NT) tissues. n = 35. (G) Kaplan-Meier survival estimate curve for TCGA-LIHC patients ranked by expression of *ARG1*. n = 135 (low), n =155 (normal). (H) Kaplan-Meier survival estimate curve for TCGA-LIHC patients ranked by expression of *AGMAT*. n = 136 (low), n = 158 (normal). (I) Polyamine species in tumors (T) relative to paired non-tumor (NT) liver tissues (log_2_ ratio). n = 11. (J) Arginine content in paired non-tumor (NT) and tumor (T) tissues of HCC patients. n = 10. (K) Total polyamine content in paired non-tumor (NT) and tumor (T) tissues of HCC patients. n = 10. (L) Volcano plot of the −log_10_(adjusted p value) against the log_2_ fold-change of 600 proteins identified by MS (in minimum 2 out of 3 samples) after purification from HCC tissues by arginine (Arg)- compared to leucine (Leu)-coupled agarose beads. Red dot highlights RBM39. (M) Dose-response curve of 20 HCC patient-derived organoids treated with sorafenib. Data are presented as the percentage of control DMSO-treated tumor organoids. (N) IC_50_ of indisulam- and sorafenib-treated HCC patient-derived organoids. n = 20. (O and P) *Rbm39* and *Asns* mRNA levels in embryonic day 14 (E14), E18, and adult mouse liver as reads per kilobase of exon per million reads mapped (RPKM). Data from NBCI Gene.^74^ n.s. = not significant, ^∗^p < 0.05, ^∗∗^p < 0.01, ^∗∗∗^p < 0.001, ^∗∗∗∗^p < 0.0001 by paired t test (A–C, J, K, and N), multiple t test (F and I), and log rank test (G and H).

We next sought to determine whether the above results, obtained in mouse tumors and human cancer cells, translate to patients. We first analyzed the proteomes and transcriptomes of biopsies obtained from liver tumors and adjacent non-tumor tissues of HCC patients.^50^ As observed in L-dKO tumors, HCC biopsies displayed suppression of the urea cycle, upregulation of several arginine transporters, and deregulation of polyamine biosynthetic enzymes. Most importantly, expression of ARG1 and AGMAT was decreased and expression of RBM39 and ASNS was increased in HCC (Figures 7A and S7A–S7D). These alterations were particularly pronounced in dedifferentiated, aggressive tumors (i.e., tumors clinically classified as Edmondson-Steiner high grade), which is consistent with the phenotype in our aggressive liver cancer mouse model. Immunoblotting confirmed loss of ARG1 and AGMAT and increased levels of RBM39 and ASNS in tumors of liver cancer patients compared to adjacent non-tumor tissue (Figure 7B). Furthermore, tissue microarray analysis of more than one hundred HCC samples confirmed significant loss of ARG1 and AGMAT in HCC (Figures 7C, 7D, and S7E). Interestingly, analysis of the transcriptome of early-stage HCC^51^ revealed downregulation of ARG1 and AGMAT and upregulation of RBM39 and ASNS (Figure S7F). This supports the hypothesis that loss of ARG1 and AGMAT, and thereby up-regulation of ASNS through RBM39, are early events in HCC. Moreover, the importance of ARG1 and AGMAT expression in HCC patients is underscored by the finding that loss of ARG1 and/or AGMAT is associated with reduced survival based on a TCGA liver cancer dataset (Figures 7E, S7G, and S7H).

We also assessed changes in arginine-related metabolites in patient samples. Untargeted metabolomics on 11 paired tumor and non-tumor patient biopsies revealed that the urea cycle metabolites ornithine and citrulline were decreased while arginine and acetylated polyamines were increased (Figures 7F and S7I). In addition, we biochemically confirmed the increase in arginine and total polyamines in an independent cohort of 10 paired tumor and non-tumor tissues (Figures S7J and S7K).

Next, we performed pull-down experiments to identify potential arginine binding proteins in human liver cancer tissues. 289 proteins were significantly enriched upon pull-down with arginine-coupled beads (Figure S7L). Importantly, RBM39 was detected as an arginine-binding protein in lysates of three out of three examined HCC samples (Figures 7G and S7L).

Finally, we treated 20 patient-derived HCC organoids^52^ with indisulam. Indisulam reduced growth in all 20 organoids in a dose-dependent manner (Figure 7H). Interestingly, the half-maximal inhibitory concentration (IC_50_) of indisulam was low compared to sorafenib, a multi-kinase inhibitor used in advanced stage HCC^53^ (Figures S7M and S7N). This suggests that depletion of RBM39 could be a therapeutic option in HCC. Altogether, the above indicates that our findings in mouse tumors and human SNU-449 cells translate to HCC patients and that the arginine-RBM39-dependence of HCC could be exploited therapeutically with aryl sulfonamides.

## Discussion

Here we show that arginine levels are increased in HCC, despite suppression of arginine synthesis, due to increased arginine import and decreased arginine-to-polyamine conversion by ARG1 and AGMAT. The high levels of arginine reprogram metabolism to promote tumorigenicity. Arginine binds RBM39 and thereby controls metabolic gene expression. Importantly, RBM39 promotes ASNS expression and thus asparagine synthesis. Asparagine further enhances arginine uptake, creating a positive feedback loop to sustain high arginine levels and oncogenic metabolism (Figure 7I).

Alterations in the arginine-synthesizing urea cycle are common in cancer.^16^^,^^17^ However, while there has been much focus on alterations in expression of urea cycle enzymes, little is known about arginine levels. In RCC, suppression of the urea cycle results in low arginine levels.^54^ In contrast, we unexpectedly observed high arginine levels despite suppression of arginine synthesis. We note that our observation of increased arginine in liver tumors has been confirmed by others.^55^

Our finding that arginine promotes oncogenic metabolism, and thus tumorigenicity, is analogous to observations in T cells. T cell activation, proliferation, and survival depend on arginine.^56^ Interestingly, arginine at supraphysiological levels reduces glycolysis and stimulates gluconeogenesis, OXPHOS, and nucleotide synthesis, possibly via arginine-binding proteins.^18^ However, T cells rapidly metabolize the excess arginine, leading to reduced arginine levels. In contrast, liver tumors reduce arginine-to-polyamine conversion to sustain high levels of unmetabolized arginine. Given the dependence of T cells on arginine, we note that increased arginine uptake by tumor cells, and thus depletion of arginine in the tumor microenvironment, might contribute to immune escape by the tumor. Tumor cells benefit from increased arginine uptake in two different ways.

We propose that RBM39 is an arginine-binding regulator of metabolic genes, including *ASNS,* in HCC. How does RBM39 control metabolic gene expression? RBM39, depending on its interacting proteins, is a pre-mRNA splicing factor or a transcription coactivator or co-repressor.^43^^,^^57^^,^^58^ Depletion of RBM39 or restriction of arginine (i.e., ARG1/AGMAT expression) impaired transcription but not splicing of metabolic genes, suggesting that RBM39 controls metabolic gene expression at the level of transcription. We observed both up- and downregulation of metabolic genes upon RBM39 depletion or arginine restriction. Thus, RBM39 might interact with transcription activators and repressors in an arginine-dependent manner. It is of high interest to identify potential transcription regulators that interact with RBM39, possibly in an arginine-dependent manner.

Our data suggest that arginine binds to the N-terminal region of RBM39, which is structurally unresolved and presumably disordered. Interestingly, the tyrosine kinase c-Abl phosphorylates residues in the N terminus to enhance transcriptional coactivation by RBM39.^59^ Thus, the N terminus of RBM39 might be an important regulatory region integrating various inputs including phosphorylation and arginine binding.

We note that *Asns* and *Rbm39* expression is high in developing, embryonic liver and low in adult liver (Figures S7O and S7P). Thus, expression of *Asns* and *Rbm39* may reflect an embryonic metabolic state that is re-activated in HCC, consistent with the notion that cancer cells are de-differentiated. Overall, the above findings suggest that arginine acts as a second messenger-like molecule in tumors and embryonic liver development.

Can arginine’s role in promoting oncogenic metabolism be exploited for cancer therapy? The observation that tumors often lack urea cycle enzymes, and are thus dependent on exogenous arginine, has led to the development of circulating arginine-degrading enzymes as a therapeutic strategy.^16^^,^^17^ However, clinical benefit related to cancer progression or patient survival has been very limited.^17^^,^^60^^,^^61^^,^^62^ Our findings suggest an alternative strategy, namely targeting a cancer-specific arginine-binding factor such as RBM39 rather than broadly limiting arginine in circulation. This would also avoid the undesirable side effect of inhibiting T cells, which require arginine for activation.^18^^,^^56^ Interestingly, molecular glues such as the aryl sulfonamide indisulam specifically target RBM39 for ubiquitination and degradation. Indeed, we have shown that treating HCC cells with indisulam mimics the effect of arginine depletion. RBM39-degrading aryl sulfonamides, like arginine-degrading enzymes, have shown little clinical benefit (see Xu et al.^43^ and references therein). However, the aryl sulfonamides have not been tested in HCC patients. Our findings in mice, cells, and patient-derived organoids suggest that HCC patients with high tumoral arginine levels (i.e., loss of ARG1 and AGMAT and gain of ASNS) and elevated RBM39 levels would benefit from treatment with aryl sulfonamides.

### Limitations of the study

Our study has revealed that high arginine levels promote metabolic reprogramming by binding to the N-terminal domain of RBM39. Follow-up studies involving structural analysis and point mutations are required to determine the precise arginine-binding site(s) in RBM39. Furthermore, (transcription) factors that interact with RBM39, possibly in an arginine-dependent manner, to promote metabolic gene expression will be of interest. Characterization of such factors and the precise arginine-binding site(s) in RBM39 may reveal the mechanism by which arginine activates RBM39. RBM39-interacting proteins may also elucidate the relationship between RBM39 and ATF4, two factors involved in *ASNS* expression. Metabolic flux analyses will reveal the extent to which arginine is metabolized in HCC and other cancers. Finally, it remains to be determined whether arginine controls metabolic gene expression in other cancers and whether other cancers could be targeted with aryl sulfonamides.

## STAR★Methods

### Key resources table

**Table:** 

REAGENT or RESOURCE	SOURCE	IDENTIFIER
**Antibodies**		

Rabbit anti-ARG1	GeneTex	Cat#109242
Rabbit anti-AGMAT	Novus Biological	Cat#1-82080
Rabbit anti-CPS1	abcam	Cat#129076
Mouse anti-OTC	SantaCruz Biotech	Cat#515791
Mouse anti-ASS1	SantaCruz Biotech	Cat#365475
Mouse anti-ASL	SantaCruz Biotech	Cat#166787
Rabbit anti-SLC7A1	abcam	Cat#37588
Rabbit anti-SLC7A6	MyBiosource	Cat#7103267
Rabbit anti-SLC7A7	Epigentek	Cat#A68118-020
Rabbit anti-ODC	GeneTex	Cat#54600
Rabbit anti-SRM	ThermoFisher Scientific	Cat#PA5-31341
Mouse anti-SMS	SantaCruz Biotech	Cat#376294
Rabbit anti-SAT1	Novus Biological	Cat#110-41622
Mouse anti-PAOX	SantaCruz Biotech	Cat#166185
Rabbit anti-SMOX	abcam	Cat# 213631
Rabbit anti-AKT	Cell Signaling	Cat#4685
Rabbit anti-AKT-pS473	Cell Signaling	Cat#9217
Rabbit anti-Calnexin	Enzo Life Sciences	Cat#ADI-SPA-860-F
Mouse anti-Actin	Millipore	Cat#MAB1501
Rabbit anti-ASNS	GeneTex	Cat#30068
Rabbit anti-PSAT1	GeneTex	Cat#633629
Rabbit anti-PSPH	GeneTex	Cat#33442
Mouse anti-NNMT	abcam	Cat#119758
Rabbit anti-S6	Cell Signaling	Cat#2217
Rabbit anti-S6-pS240,244	Cell Signaling	Cat#5364
Rabbit anti-RBM39	Sigma	Cat#HPA001591
Rabbit anti-RBM39	Bethyl Laboratories	Cat#A300-291A
Mouse anti-FLAG M2	Sigma	Cat#F1804
Mouse anti-HA	Cell Signaling	Cat#2367
Mouse anti-Strep	Invitrogen	Cat#MA5-37747
Mouse anti-eIF2α	Cell Signaling	Cat#2103
Rabbit anti-eIF2α-pS51	Cell Signaling	Cat#3957
Rabbit anti-SESN2	abcam	Cat#ab178518
Rabbit anti-AGMAT (for IHC)	Sigma	Cat#PA5-55311
Mouse anti-CASTOR1	SantaCruz Biotech	Cat#377114
Mouse anti-H3	Cell Signaling	Cat#14269
Mouse anti-GAPDH	SantaCruz Biotech	Cat#365062

**Biological samples**		

HCC patient tumor and non-tumor tissues	University Hospital Basel	N/A
HCC patient-derived organoids	University Hospital Basel	N/A

**Chemicals, peptides, and recombinant proteins**		

Indisulam	Sigma	Cat#SML1225
Indisulam	MedKoo Biosciences	Cat#201540
L-Arginine-coupled agarose beads	gbiosciences	Cat#GENO786-1361
L-leucine-coupled agarose beads	gbiosciences	Cat#GENO786-1370

**Critical commercial assays**		

Total Polyamine Assay Kit	BioVision	Cat#K475
L-arginine ELISA kit	MyBiosource	Cat#MBS728648-96
Nano-Glo dual luciferase reporter assay kit	Promega	Cat#N1610

**Deposited data**		

HCC patients’ RNA sequencing data	Ng et al., 2022^50^	EGAS00001005073, EGAS00001005074
HCC patients’ proteome data	Ng et al., 2022^50^	PRIDE (PXD025705, PXD025836)
Mouse RNA sequencing data	Dimitrakopoulos et al., 2021^25^	SRP156216
Mouse proteome data	Dimitrakopoulos et al., 2021^25^	https://github.com/cbgethz/netics/tree/master/mouse_data
Mouse and HCC patients’ metabolomics	This paper	MASSIVE: MSV000092406
RNA sequencing data of ARG1/AGMAT-expressing (and control) and RBM39-depleted (and control) SNU-449 cells	This paper	GEO: PRJNA940402
Mouse, HCC patients, and SNU-449 proteome data of pulldowns using Leu- and Arg-coupled agarose beads	This paper	MASSIVE: MSV000091516

**Experimental models: Cell lines**		

SNU-182	Diego Calvisi (University of Sassari, Italy)	N/A
SNU-449	Diego Calvisi (University of Sassari, Italy)	N/A
HLE	Diego Calvisi (University of Sassari, Italy)	N/A
PLC	Diego Calvisi (University of Sassari, Italy)	N/A
Hep40	Diego Calvisi (University of Sassari, Italy)	N/A
Hep3B	Diego Calvisi (University of Sassari, Italy)	N/A
Huh6	Diego Calvisi (University of Sassari, Italy)	N/A
Huh7	Diego Calvisi (University of Sassari, Italy)	N/A
HepG2	Diego Calvisi (University of Sassari, Italy)	N/A
SNU-475	Gerhard Christofori (University of Basel, Switzerland)	N/A
SNU-423	Gerhard Christofori (University of Basel, Switzerland)	N/A
Huh1	Gerhard Christofori (University of Basel, Switzerland)	N/A
HEK293	ATCC	CRL-1573
HEK239T	ATCC	CRL-3216
SNU-449 control	This paper	N/A
SNU-449 ARG1	This paper	N/A
SNU-449 AGMAT	This paper	N/A
SNU-449 ARG1/AGMAT	This paper	N/A
SNU-449 ASS1-FLAG	This paper	N/A
SNU-449 ARG1/AGMAT +ASS1	This paper	N/A
SNU-449 ARG1/AGMAT +3xHA-ASS1	This paper	N/A
Huh7 sgCtrl	This paper	N/A
Huh7 sgARG1	This paper	N/A
Huh7 sgAGMAT	This paper	N/A
SNU-449 ARG1/AGMAT +control (for ASNS)	This paper	N/A
SNU-449 ARG1/AGMAT +ASNS	This paper	N/A
SNU-449 ARG1/AGMAT +3xHA-RBM39	This paper	N/A
SNU-449 ARG1/AGMAT +control (for 3xHA-RBM39)	This paper	N/A
SNU-449 shCtrl	This paper	N/A
SNU-449 sh*RBM39*_1	This paper	N/A
SNU-449 sh*RBM39*_2	This paper	N/A
SNU-449 RBM39(1–529; G268V)-FLAG (termed full-length)	This paper	N/A
SNU-449 RBM39(Δ11–132; G268V)-FLAG (termed ΔN)	This paper	N/A
SNU-449 RBM39(Δ11–132; cMYC-NLS; G268V)-FLAG (termed ΔN-NLS^cMYC^)	This paper	N/A

**Experimental models: Organisms/strains**		

Mouse: B6.129S4-Tsc1<tm1Djk> x B6.129S4-Pten<tm1Hwu>/J x B6.Cg-Tg(Alb-cre)21Mgn/J (L-dKO)	Guri et al. 2017^23^; Hindupur et al. 2018^25^	N/A
Mouse: B6.129S4-Tsc1<tm1Djk> x B6.129S4-Pten<tm1Hwu>/J x B6.Cg-21Mgn/J (Ctrl)	Guri et al. 2017^23^; Hindupur et al. 2018^25^	N/A

**Oligonucleotides**		

PCR and qPCR primers: Table S5	Harvard Primer Bank/This paper	N/A
Site-directed mutagenesis (SDM) primers: Table S5	This paper	N/A
siRNA: custom-made siRNA screen	Horizon Discovery/This paper	N/A
siRNA: ON-TARGETplus Human RBM39 siRNA	Horizon Discovery	J-011965-06-0020
CRISPR: sgARG1: fw: CACCGTCCAATAATCCCT ATGGTTC, rv: AAACGAACCATAGGGATTATTGGAC	This paper	N/A
CRISPR: sgAGMAT: fw: CACCGACCGGCCGGGC CAC GAACTC, rv: AAACGAGTTCGTGGCCCGGC CGGTC	This paper	N/A

**Recombinant DNA**		

pLenti-GIII-CMV-ARG1:GFP-2A-Puro	abmgood	Cat#LV078906
pLenti-GIII-CMV-AGMAT::RFP-2A-Puro	abmgood	Cat#LV071242
pLenti-CMV-GFP-2A-Puro-Blank Vector	abmgood	Cat#LV590
pReceiver-Lv197-CMV-ASS1	genecopoeia	Cat#EX-C0719-Lv197
pLenti-CMV-ASS1-C-Myc-DDK-P2A-Puro	Origene	Cat#RC201130L3
pReceiver-Lv118-CMV-ASS1-N-3xHA	genecopoeia	Cat#EX-C0719-Lv118
pLenti-CMV-ASNS	genecopoeia	Cat#EX-C0302-Lv197
pLenti-CMV-Empty control vector (for ASNS)	genecopoeia	Cat#EX-NEG-Lv197
psPAX2	gift from Didier Trono	Addgene #12260
pCMV-VSV-G	gift from Robert Weinberg	Addgene #8454
pLentiCRISPRv2	Sanjana et al., 2014^63^	Addgene #52961
pReceiver-Lv118-CMV-RBM39-N-3xHA	genecopoeia	Cat#EX-Z5354-Lv118
pReceiver-Lv118 Empty control vector (for 3xHA-RBM39)	genecopoeia	Cat#EX-NEG-Lv118
pCMV6-CASTOR1-Myc-DDK	Origene	Cat#RC205868
pCMV6-RBM39-Myc-DDK	Origene	Cat#RC224584
pLenti-eGFP-FLAG	Lab stock	N/A
pLenti-CMV-RBM39-C-Myc-DDK-P2A-Puro	Origene	Cat#RC224584L3
pNL1.1.TK[Nluc/TK]	Promega	Cat#PAN1501
pGL4.10[luc2]-ASNS promoter region	Genscript/This paper	N/A
pGL4.10[luc2]-PSAT1 promoter region	Genscript/This paper	N/A
shRNA: pLenti-shRBM39	Sigma Aldrich	TRCN0000232612
shRNA: pLenti-sh non-targeting control	Sigma Aldrich	SHC002
pLenti-CMV-RBM39(G268V)-C-Myc-DDK-P2A-Puro	This paper	N/A
pLenti-CMV-RBM39(Δ11–132; G268V)-C-Myc-DDK-P2A-Puro	This paper	N/A
pLenti-CMV-RBM39(Δ11–132; cMYC-NLS; G268V)-C-Myc-DDK-P2A-Puro	This paper	N/A
pETG-10K	Gift from Timm Maier	N/A
pETG-10K-RBM39-C-Strep	This paper	N/A
pETG-10K-RBM39(1–244)-C-Strep	This paper	N/A
pETG-10K-RBM39(143–529)-C-Strep	This paper	N/A
pETG-10K-RBM39(245–529)-C-Strep	This paper	N/A

**Software and algorithms**		

GraphPad Prism 9	https://www.graphpad.com/scientific-software/prism/	N/A
Fiji	https://fiji.sc/#cite	N/A
ZEN 2 (blue edition)	Carl Zeiss	N/A
Tecan i-control, version 1.11.1.0	Tecan	N/A
NxtIRFcore	https://github.com/alexchwong/NxtIRFcore	N/A

**Other**		

Mouse diet with 100% arginine	ssniff Spezialdiäten	N/A
Mouse diet with 10% arginine	ssniff Spezialdiäten	N/A
Mouse diet with 1% arginine	ssniff Spezialdiäten	N/A
AAV8-hAAT-null (AAV-Ctrl)	This paper	N/A
AAV8-hAAT-mARG1 (AAV-ARG1)	This paper	N/A
AAV8-hAAT-mAGMAT (AAV-AGMAT)	This paper	N/A
AAV-DJ[ssAAV.ALB.EGFP.miR30shRNA(mScramble).WPRE.SV40pA] (AAV-shCtrl),	Packgene/This paper	N/A
AAV-DJ[ssAAV.ALB.EGFP.miR30shRNA(mASNS).WPRE.SV40pA] (AAV-sh*Asns*)	Packgene/This paper	N/A
AAV-DJ[ssAAV.ALB.EGFP.miR30shRNA(mBM39).WPRE.SV40pA] (AAV-sh*Rbm39*)	Packgene/This paper	N/A

### Resource availability

#### Lead contact

Requests for information and reagents should be directed to and will be fulfilled by the lead contact, Michael N. Hall (m.hall@unibas.ch).

#### Materials availability

All unique/stable reagents and data generated in this study are available from the lead contact without restriction.

### Experimental model and study participant details

#### Animals

Liver-specific Tsc1 and Pten double-knockout mice (L-dKO) on mixed genetic background (C57BL/6J, 129/SvJae, BALB/cJ) were as described.^23^^,^^24^^,^^25^ Male animals (Cre-positive (L-dKO) and Cre-negative littermates as control (Ctrl)) were used for experiments. Mice were housed at 22°C with a 12 h light/dark cycle with unlimited access to water and food. In all experiments, mice were fasted overnight before euthanasia by CO_2_ inhalation. All animal experiments were performed in accordance with the federal ethical guidelines and were approved by the Kantonales Veterinäramt of Basel-Stadt (Licenses: 2965_29711, 2965_33264).

#### Human cell lines

Human liver cancer cell lines SNU-182, SNU-449, HLE, PLC, Hep40, Hep3B, Huh6, Huh7, and HepG2 were gifted by Prof. Diego Calvisi (University of Greifswald, Germany), SNU-475, SNU-423, and Huh1 were gifted by Prof. Gerhard Christofori (University of Basel, Switzerland). HEK293 and HEK293T cells were obtained from ATCC. All cells, except Huh1, were cultured in high glucose-containing DMEM (Sigma, Cat# D5671) supplemented with 10% FBS, 2 mM glutamine, 0.1 mM non-essential amino acids (Gibco, Cat# 11140-035), and 1X penicillin/streptomycin (hereafter, DMEM complete). Huh1 cells were cultured in low glucose-containing DMEM (Sigma, Cat# D6046) supplemented with 10% (v/v) FBS, 0.1 mM non-essential amino acids (Gibco, Cat# 11140-035), and 1X penicillin/streptomycin. For experiments involving modification of arginine concentration in the medium, cells were cultured in DMEM lacking arginine and lysine (ThermoFisher Scientific, Cat# 88364) supplemented with 10%(v/v) dialyzed FBS (ThermoFisher Scientific, Cat# 26400044), 0.798 mM lysine, 1X penicillin/streptomycin, 0.1 mM non-essential amino acids (Gibco, Cat# 11140-035) and 3.98 μM of arginine (1% compared to standard DMEM medium), or as indicated. Cells were incubated at 37°C with 5% CO_2_ and tested for mycoplasma on regular basis.

#### Patient material and ethics

All relevant ethical regulations were followed in this study. Human tissues were obtained from patients undergoing diagnostic liver biopsy at the University Hospital Basel between 2008 and 2018. Written informed consent was obtained from all patients. The study was approved by the ethics committee of Northwestern Switzerland (EKNZ, approval No. 2014-099). Ultrasound-guided needle biopsies were obtained from tumor lesion(s) and the liver parenchyma at a site distant from the tumor with a coaxial liver biopsy technique that allows taking several biopsy samples through a single biopsy needle tract as described.^50^^,^^52^ Clinical disease staging was performed using the Barcelona Clinic Liver Cancer system.^64^ In total, 122 HCC biopsies and 115 non-tumoral tissues from 114 patients with different disease etiologies were included in the study.^50^ The ethics committee of Northwestern Switzerland approved all the experiments with resected human tissue samples which were used for immunoblotting and biochemical assessment of arginine and polyamine levels reported in this study (EKNZ, approval No. 361/12).

For identification of potential arginine binding proteins, HCC samples were obtained from three patients undergoing surgery at the University Center for Gastrointestinal and Liver Disease (Clarunis), Basel, Switzerland. Written informed consent was obtained from all patients. The study was performed in accordance with the Helsinki Declaration and approved by the ethics committee (Ethics Committee of Basel, EKBB, No. 2019–02118). Age, sex and other patient characteristics available are described in Ng et al.^50^ We did not have access to information related to ancestry and socioeconomic status.

#### HCC organoid culture

HCC tissues for organoid generation were collected from patients undergoing diagnostic liver biopsy or surgical resection at the University Hospital Basel. Written informed consent was obtained from all patients. The study was approved by the local ethics committee (protocol numbers EKNZ 2014-099 and BASEC 2019-02118). HCC organoids (HCCOs) were generated as described previously.^52^ Briefly, tissues were dissociated to small cell clusters and seeded into basement membrane extract type 2 (BME2, R&Dsystems, Cat# 3533-005-02). After BME2 polymerization, expansion medium (EM) was added. The EM composition is as follows: advanced DMEM/F-12 (Gibco, Cat# 12634010) supplemented with B-27 (Gibco, Cat# 17504001), N-2 (Gibco, Cat# 17502001), 10 mM Nicotinamide (Sigma, Cat# N0636), 1.25 mM N-acetyl-L-cysteine (Sigma, Cat# A9165), 10 nM [Leu^15^]-Gastrin (Sigma, Cat# G9145), 10 μM Forskolin (Tocris, Cat# 1099), 5 μM A83-01 (Tocris, Cat# 2939), 50 ng/mL EGF (Peprotech, Cat# AF-100-15), 100 ng/mL FGF10 (Peprotech, Cat# 100-26), 25 ng/mL HGF (Peprotech, Cat# 100-39), 10% RSpo1-conditioned medium (v/v, homemade).

### Method details

#### Arginine-modified diets

For diet experiments mice were fed with arginine-modified diets from 8 to 20 weeks of age. Diets were purchased from ssniff Spezialdiäten (Germany) and differed in arginine content (100% corresponding to the concentration of arginine contained in the standard diet of the animal facility (Kliba 3436)). Differences in protein/nitrogen content were balanced by increased concentrations of glycine and alanine.

#### AAV administration

For AAV administration, 7-8 weeks-old Ctrl and L-dKO animals were infected via tail vein injection. For expression of ARG1 and AGMAT, AAV8-hAAT-null (AAV-Ctrl), AAV8-hAAT-mARG1 (AAV-ARG1), or AAV8-hAAT-mAGMAT (AAV-AGMAT) were injected at 2x10ˆ12 genome copies (GC) in PBS/0.001% Pluronics F68 per mouse. AAV particles were provided by Prof. Fatima Bosch (Universitat Autònoma de Barcelona, Spain) and underlying constructs (AAV8-hAAT) were described previously.^34^ For knockdown of *Asns* and *Rbm39* following AAV particles were purchased from Packgene (USA): AAV-DJ[ssAAV.ALB.EGFP.miR30shRNA(mScramble).WPRE.SV40pA] (AAV-shCtrl), AAV-DJ[ssAAV.ALB.EGFP.miR30shRNA(mASNS).WPRE.SV40pA] (AAV-sh*Asns*), AAV-DJ[ssAAV.ALB.EGFP.miR30shRNA(mRBM39).WPRE.SV40pA] (AAV-sh*Rbm39*). 5x10ˆ11 GC in PBS were injected per mouse.

#### Indisulam treatment of mice

Indisulam (MedKoo Biosciences, Cat# 201540) was dissolved at 75 mg/mL in DMSO.

37.5 mg indisulam per kg body weight, prepared in vehicle (5% (v/v) Tween80 in 0.9% (w/v) NaCl), was administered via intraperitoneal injection at 10 mL/kg body weight. 16 weeks-old L-dKO mice were injected seven times with indisulam or vehicle over a period of 2 weeks.

#### HCC organoid drug treatment

Sorafenib tosylate (Selleckchem, Cat# S1040) and Indisulam (Sigma, Cat# SML1225) were dissolved in DMSO at 100 mM and stored in 10 mM aliquots. For drug sensitivity assays 20 different HCCO lines were used. Each HCCO line was dissociated into single cells using 0.25% Trypsin-EDTA (Gibco, Cat# 25200056) and plated at 1′000 cells/well in a 384-well plate. After 3 days, drugs were added using a D300e digital dispenser (Tecan) and the HCCOs treated for a total of 5 days. Cell viability was measured using the CellTiter-Glo 3D reagent (Promega) according to the manufacturer’s instructions (G9681). Results were normalized to vehicle (DMSO) and curve fitting was performed using Prism v9.5.1 (GraphPad) software and the nonlinear regression fitting (four parameters model).

#### Generation of stable cell lines

For stable expression of ARG1 and/or AGMAT, ARG1 lentiviral vector (pLenti-GIII-CMV-ARG1:GFP-2A-Puro, Cat# LV078906), AGMAT lentiviral vector (pLenti-GIII-CMV-AGMAT::RFP-2A-Puro, Cat# LV071242), or Control lentiviral vector (pLenti-CMV-GFP-2A-Puro-Blank Vector, Cat# LV590) were purchased from abmgood (Canada). For stable expression of ASS1, lentiviral vectors (pReceiver-Lv197-CMV-ASS1, Cat#EX-C0719-Lv197 and pReceiver-Lv118-CMV-ASS1-N-3xHA, Cat#EX-C0719-Lv118) were purchased from genecopoeia (USA) and lentiviral vector (pLenti-CMV-ASS1-C-Myc-DDK-P2A-Puro, Cat#RC201130L3) was purchased from Origene. For stable expression of ASNS, lentiviral vector (pLenti-CMV-ASNS, Cat# EX-C0302-Lv197) or Control lentiviral vector (pLenti-CMV-Empty control vector, Cat# EX-NEG-Lv197) were purchased from genecopoeia (USA). For stable expression of 3xHA-RBM39, lentiviral vector (pReceiver-Lv118-CMV-RBM39-N-3xHA, Cat# EX-Z5354-Lv118) or Control lentiviral vector (pReceiver-Lv118 Empty control vector, Cat# EX-NEG-Lv118) were purchased from genecopoeia (USA). For stable knockdown of *RBM39*, shRBM39 lentivral vector (Cat# TRCN0000232612) or Control non-targeting lentiviral vector (Cat# SHC002) were purchased from Sigma Aldrich. For CRISPR/Cas9-mediated knockout, guide RNA for ARG1 (fw: CACCGTCCAATAATCCCTATGGTTC, rv:AAACGAACCATAG GGATTATTGGAC) and AGMAT (fw: CACCGACCGGCCGGGCCACGAACTC, rv: AAACGAGTTCGTGGCCCGGCCGGTC) were cloned into pLentiCRISPRv2^63^ (Addgene #52961). Non guide RNA-containing vector served as control.

For the stable expression of indisulam-resistant RBM39 a G268V mutation was generated on pLenti-CMV-RBM39-C-Myc-DDK-P2A-Puro (purchased from Origene, Cat# RC224584L3) using site-directed mutagenesis (SDM). The resulting plasmid (pLenti-CMV-RBM39(G268V)-C-Myc-DDK-P2A-Puro) was subsequently used as a template and the N-terminus (residues 11–132) was deleted by SDM to generate pLenti-CMV-RBM39(Δ11–132; G268V)-C-Myc-DDK-P2A-Puro. Subsequently, the NLS of the oncoprotein MYC (PAAKRVKLD) was inserted after residue 10 using SDM yielding pLenti-CMV-RBM39(Δ11–132; cMYC-NLS; G268V)-C-Myc-DDK-P2A-Puro. Primer pairs for SDM were as listed in Table S5.

HEK293T cells were co-transfected with a lentiviral vector, psPAX2 (a gift from Didier Trono: Addgene plasmid # 12260) and pCMV-VSV-G (a gift from Robert Weinberg: Addgene plasmid # 8454) using jetPRIME from Polyplus (France). Supernatants containing lentiviral particles were collected and filtered 48 h after transfection. Filtered supernatants were used to infect SNU-449 cells in the presence of 10 μg/mL polybrene. Infected cells were selected with 2 μg/mL puromycin, 20 μg/mL blasticidin, or 1 mg/mL G418, depending on the selection marker of the used lentiviral plasmid. To obtain clones with similar expression levels in single (ARG1 or AGMAT) and double (ARG1 and AGMAT) expressing cells, puromycin-selected cells were single cell-sorted by FACS for GFP (Ctrl and ARG1), RFP (AGMAT), or GFP and RFP (ARG1 and AGMAT) and expression of ARG1 and AGMAT was tested by immunoblotting.

#### Growth under arginine restricted conditions and indisulam treatment

SNU-449 cells were seeded in DMEM complete medium. Medium was exchanged the following day (to medium restricted to 1% arginine) and cells were incubated for 5–7 days. Indisulam (Sigma, Cat# SML1225) was dissolved in DMSO at 100 mM. Cells were treated with 10 μM indisulam (or equivalent volumes of DMSO) for either 2 days daily (for RNA-seq, in combination with siRNA-mediated knockdown of RBM39, see below), 3–5 days daily (for deep RBM39 depletion followed by immunoblot or qPCR analyses), or 5–14 days every 3 days (for clonogenic growth assays) in arginine-restricted medium.

#### siRNA-mediated knockdown

For siRNA-mediated knockdown, SNU-449 cells were seeded in DMEM complete medium and transfected with 100 nM SMARTpool siRNA using DharmaFECT4, both from Horizon Discovery (UK), in OptiMEM. Medium was exchanged the following day (to medium with 1% arginine) and cells were incubated for 48 h.

#### Luciferase reporter assay

10ˆ4 SNU-449 cells (control and ARG1/AGMAT or parental cell treated with DMSO or indisulam) were seeded in 96-well plates in arginine-restricted medium and transfected with 49.5 ng firefly pGL4.10[luc2] luciferase reporter vector containing a 1000bp promoter region of *ASNS* or *PSAT1* together with 0.5 ng pNL1.1.TK[Nluc/TK] internal control vector using X-tremeGENE360 (Roche). Reporter (i.e., promoter) activity was assessed using the Nano-Glo dual luciferase reporter assay kit according to the manufacturer’s instructions 48 h post-transfection. Luciferase activity was normalized by using the pNL1.1.TK[Nluc/TK] internal control.

#### Clonogenic growth assays and crystal violet staining

Low numbers of SNU-449 cells (500–2000 cells in 12-well plates; 1000–2500 cells in 6-well plates) were seeded in DMEM complete medium. Medium was exchanged the following day (to medium with 1% arginine) and cells were incubated for 5–14 days, in the presence of metabolites, if indicated. To visualize colony formation ability, cells were stained with crystal violet (2% (v/v) crystal violet in 20% (v/v) methanol). Cell growth was quantified using ImageJ.

#### Hepatosphere formation assay

1x 10ˆ5 SNU-449 cells were seeded in ultra-low attachment six-well plates (Corning, Cat# 3471) in arginine-restricted medium. Hepatosphere formation was assessed by microscope after 7 days (AxioVision and ZEN2(blue edition), Carl Zeiss).

#### Cellular fractionation

Cellular fractionation was carried out by the REAP method.^65^ In brief, cells were lysed with ice-cold 0.1% NP40-PBS, 1/3 was removed as whole cell lysate (WCL). The remaining lysate was centrifuged and 1/3 of the supernatant was removed as cytoplasmic fraction (Cyto). The nuclear pellet (Nuc) was again washed with ice-cold 0.1% NP40-PBS. All fractions were resuspended in sample buffer to the same final volume, boiled and equal volumes were analyzed by immunoblotting.

#### Immunoblots

Human and mouse liver tissues were homogenized in T-PER (ThermoFisher Scientific, Cat# 78510) supplemented with cOmplete inhibitor cocktail (Roche) and PhosSTOP (Roche) using a Polytron (PT 10–35 GT) and subsequent sonication (Hielscher UP200St). Human liver cancer cells were lysed in M-PER (ThermoFisher Scientific, Cat# 78501) supplemented with cOmplete inhibitor cocktail (Roche) and PhosSTOP (Roche). Protein concentration was determined by Pierce BCA assay (ThermoFisher Scientific, Cat# 23225), and equal amounts of protein were separated by SDS-PAGE, and transferred onto nitrocellulose membranes (GE Healthcare). Antibodies used in this study were as follows: ARG1 (GeneTex, Cat# 109242), AGMAT (Novus Biological, Cat# 1–82080), CPS1 (abcam, Cat# 129076), OTC (SantaCruz Biotech, Cat# 515791), ASS1 (SantaCruz Biotech, Cat# 365475), ASL (SantaCruz Biotech, Cat# 166787), SLC7A1 (abcam, Cat# 37588), SLC7A6 (MyBiosource, Cat# 7103267), SLC7A7 (Epigentek, Cat# A68118-020), ODC (GeneTex, Cat# 54600), SRM (ThermoFisher Scientific, Cat# PA5-31341), SMS (SantaCruz Biotech, Cat# 376294), SAT1 (Novus Biological, Cat# 110–41622), PAOX (SantaCruz Biotech, Cat# 166185), SMOX (abcam, Cat# 213631), AKT (Cell Signaling, Cat# 4685), AKT-pS473 (Cell Signaling, Cat# 9217), Calnexin (Enzo Life Sciences, Cat# ADI-SPA-860-F), Actin (Millipore, Cat# MAB1501), ASNS (GeneTex, Cat# 30068), PSAT1 (GeneTex, Cat# 633629), PSPH (GeneTex, Cat# 33442), NNMT (abcam, Cat# 119758), S6-pS240,244 (Cell Signaling, Cat# 5364), S6 (Cell Signaling, Cat# 2217), RBM39 (Sigma, Cat# HPA001591), RBM39 (Bethyl Laboratories, Cat# A300-291A), FLAG M2 (Sigma, Cat# F1804), HA (Cell Signaling, Cat# 2367), Strep (Invitrogen, Cat# MA5-37747), eIF2α (Cell Signaling, Cat# 2103), eIF2α-pS51 (Cell Signaling, Cat# 3957), SESN2 (abcam, Cat# ab178518), CASTOR1 (SantaCruz Biotech, Cat# 377114), H3 (Cell Signaling, Cat# 14269), GAPDH (SantaCruz Biotech, Cat# 365062).

#### RNA isolation, quantitative reverse transcription and endpoint PCR

Total RNA was isolated with RNeasy Kit (QIAGEN). RNA was reverse transcribed using iScript cDNA Synthesis Kit (Bio-Rad). Quantitative real-time PCR analysis was performed using Fast SYBR Green (Applied Biosystems) and q^3^TOWER (Analytik Jena). Relative expression levels were determined by normalizing each Ct value to *ACTIN* using the ΔCt method. For each gene at least three independent biological replicates were used. Endpoint PCR was used to assess *TRIM27* exon skipping. 100 ng cDNA was amplified with 5x FIREPol (Solis BioDyne) and analyzed on a 2% (w/v) agarose gel. The primer pairs were as listed in Table S5 (mouse-specific primers are annotated with “m” before the target gene name).

#### Metabolomics of mouse liver tissues and human liver biopsies

Snap-frozen liver samples from Ctrl and tumor tissues from L-dKO mice were collected and weighed (Ctrl: n = 5, weight mean = 53.7 mg, stdev = 4.2 mg; L-dKO: n = 6, weight mean = 55 mg, stdev = 11.4). Sample weights did not differ significantly between the two groups (two-tailed t-test, unequal variances p value = 0.81). Snap-frozen paired tumor and non-tumor biopsies from HCC patients were weighed and 2 μL/μg extraction buffer (acetonitrile, methanol, ddH_2_O; 2:2:1) was added. Metabolite extraction was performed as previously described.^66^ Tissue samples were kept on dry ice and homogenized in 1 mL of 70% (v/v) ethanol using a Tissue Lyser 2 (Qiagen) with a stainless steel bead at maximum speed for 1 min. Metabolites were extracted from the homogenized samples by adding 7 mL of 70% (v/v) ethanol heated to 75°C for 2 min and subsequently cooled in ice water. Extracts were separated from cell debris by centrifuging at 2,500 xg at 4°C for 10 min, dried in a SpeedDry Vacuum Concentrator (Christ), and resuspended in double-distilled water (ddH_2_O) corresponding to the measured weight, and then diluted 1:10 in ddH_2_O prior to mass spectrometric analysis.

Untargeted metabolomics were performed by flow injection analysis on an Agilent 6550 quadrupole instrument time-of-flight mass mass spectrometer as described previously.^22^ The instrument was operated in positive and negative mode (separate measurements), high-resolution (4GHz) mode. The injection sequence of samples was randomized, and all samples were injected in duplicates. Mass spectrometry data were pre-processed to collapse the time dimension, centroided, and merged into a single data matrix. Based on their accurate mass and the Human Metabolome Database reference list, ions were annotated, allowing tolerance of 0.003 amu and multiple common ESI adducts for initial metabolic pathway enrichment analysis (MPWEA). For subsequent analysis and individually presented metabolites in this study, annotated ions were then filtered for H^+^ adducts allowing tolerance of 0.001 amu.

Amino acid profiling was performed by targeted metabolomics using amino acid standards from same Ctrl liver and L-dKO tumor tissues using HILIC chromatography coupled to a 5500 QTRAP triple-quadrupole mass spectrometer in positive mode with MRM scan type as described in.^67^

#### Statistical analysis untargeted metabolomics data

The intensity data of the untargeted metabolomics analysis was processed using Perseus software (version 1.6.5.0).^68^ Log_2_-transformed technical duplicates were averaged and subsequently normalized by median subtraction. Separation between conditions was visualized using Perseus’s built-in principle component analysis (PCA) function. Significant deregulated metabolites were determined and visualized with the volcano plot function. Thresholds were set to FDR = 0.05 and S0 = 0.1, respectively. Unsupervised hierarchical clustering was performed after z-scoring across all rows without grouping. Spearman correlation was used to calculate the row and column tree distances. Maximum number of clusters was set to 300, iterations to 100 and restarts to 10. MPWEA was performed using the hierarchical clustering function with binarized data based on the volcano plot. Binarization was performed by defining increased metabolites as +1, decreased metabolites as −1 and unchanged metabolites as 0 following a randomization by 0.1. The pathway annotations were extracted from Small Molecule Pathway Database (SMPDB, update 2020) and linked to the measured metabolites in a FileMaker database as semicolon separated values, which could be imported to Perseus as a categorical column.

#### Arginine enzyme-linked immunosorbent assay(ELISA)

Arginine levels in mouse or human tissues, mouse plasma or tumor interstitial fluid (TIF), or cellular lysates were measured by L-arginine ELISA kit (MyBiosource, Cat# MBS728648-96) according to the manufacturer’s instructions. TIF was prepared as previously described.^36^

#### Total polyamine measurement

Tissue or cellular lysate total polyamine levels were measured by the fluorometric Total Polyamine Assay Kit (BioVision, Cat# K475) according to the manufacturer’s instructions.

#### ^3^H-arginine and ^3^H-putrescine uptake

For *ex vivo* uptake, freshly isolated Ctrl liver or L-dKO tumor tissues were incubated in 200 μL DMEM lacking arginine and 1 μCi L-[2,3,4-^3^H]-Arginine (American Radiolabeled Chemicals, Cat# 1421) or 0.5 μCi Putrescine [2,3-^3^H(N)] dihydrochloride (American Radiolabeled Chemicals, Cat# 0279) for 30 min at 37°C, washed twice with cold PBS and lysed with SOLVABLE (PerkinElmer, Cat# 6NE9100). For *in vitro* uptake, cells were cultured in DMEM medium containing 1% arginine for 24 h before addition of 1 μCi L-[2,3,4-^3^H]-Arginine for 60 min at 37°C. For pre-loading of cells, 100 μM asparagine or glutamine were added to the media 30 min prior to the addition of labeled arginine. Cells were washed twice with cold PBS and lysed with 1 M HCl. Intracellular ^3^H-arginine or ^3^H-putrescine was measured with a scintillation counter.

#### Pulldown of potential arginine-binding proteins from SNU-449 cells, mouse L-dKO, and human liver tumor tissues

SNU-449 cells were seeded in 15 cm plates in DMEM complete medium. At 80–90% confluency, cells were starved for arginine for 16h prior to harvest. SNU-449 cells, L-dKO tumor tissues, and human resected tumor tissues were lysed in buffer 1 (1% (v/v) IGEPAL-CA630, 150 mM KCl, 1 mM MgCl_2_, 0.2 mM CaCl_2_, 1 mM EDTA, 1 mM DTT, 10% (v/v) glycerol, cOmplete inhibitor cocktail (Roche) and PhosSTOP (Roche), 20 mM HEPES-NaOH pH 7.9). Protein concentration was determined by BCA assay. L-Arginine (Arg)- and L-leucine (Leu)-coupled agarose beads were purchased from gbiosciences (USA) (Arg Cat# GENO786-1361, Leu Cat# GENO786-1370). 30 μL Arg- or Leu-coupled agarose beads were equilibrated twice with 1 mL buffer 1. Binding of 2 mg of protein to equilibrated beads was allowed for 4 h at 4°C and 15 rpm rotation. Beads were washed three times with buffer 2 (0.1% (v/v) IGEPAL-CA630, 150 mM KCl, 1 mM MgCl_2_, 0.2 mM CaCl_2_, 1 mM EDTA, 1 mM DTT, cOmplete inhibitor cocktail (Roche) and PhosSTOP (Roche), 1 mM L-arginine, 10 mM L-lysine, 100 mM L-leucine, 20 mM HEPES-NaOH pH 7.9) followed by three washes with buffer 3 (150 mM KCl, 1 mM MgCl_2_, 0.2 mM CaCl_2_, 1 mM EDTA, 1 mM DTT, cOmplete inhibitor cocktail (Roche) and PhosSTOP (Roche), 1 mM L-arginine, 10 mM L-lysine, 100 mM L-leucine, 20 mM HEPES-NaOH pH 7.9). Bound proteins were eluted in 30 μL 20 mM Tris pH 8.1 containing 500 mM L-arginine. 50% (v/v) of elution fractions were prepared for MS analysis (see below) and 50% (v/v) were analyzed by immunoblotting or silver staining.

#### Identification of potential arginine-binding proteins from SNU-449 cells, mouse L-dKO, and human liver tumor tissues by MS

Eluates (see above) were brought to 5% SDS, 0.1 M TEAB. Eluted proteins were reduced in 10 mM TCEP for 10 min at 95°C, alkylated in 20 mM Iodoacetamide for 30 min at 25°C and were digested using S-Trap micro spin columns (Protifi) according to the manufacturer’s instructions. Shortly, 12% phosphoric acid was added to each sample (final concentration of phosphoric acid 1.2%) followed by the addition of S-trap buffer (90% methanol, 100 mM TEAB pH 7.1) at a ratio of 6:1. Samples were mixed by vortexing and loaded onto S-trap columns by centrifugation at 4000 xg for 1 min followed by three washes with S-trap buffer. Digestion buffer (50 mM TEAB pH 8.0) containing sequencing-grade modified trypsin (1/25 (w/w); Promega (USA)) was added to the S-trap column and incubate for 1h at 47°C. Peptides were eluted by the consecutive addition and collection by centrifugation at 4000 xg for 1 min of 40 μL digestion buffer, 40 μL of 0.2% formic acid and finally 35 μL 50% acetonitrile, 0.2% formic acid. Samples were dried under vacuum and stored at −20°C until further use.

Dried peptides were resuspended in 0.1% aqueous formic acid and subjected to LC-MS/MS analysis using a Q Exactive HF Mass Spectrometer fitted with an EASY-nLC 1000 (both Thermo Fisher Scientific) and a custom-made column heater set to 60°C. Peptides were resolved using an RP-HPLC column (75 μm × 30 cm) packed in-house with C18 resin (ReproSil-Pur C18–AQ, 1.9 μm resin; Dr. Maisch GmbH) at a flow rate of 0.2 μL min^−1^. The following gradient was used for peptide separation: from 5% B to 10% B over 5 min to 35% B over 45 min to 50% B over 10 min to 95% B over 2 min followed by 18 min at 95% B. Buffer A was 0.1% formic acid in water and buffer B was 80% acetonitrile, 0.1% formic acid in water.

The mass spectrometer was operated in DDA mode with a total cycle time of approximately 1 s. Each MS1 scan was followed by high-collision-dissociation (HCD) of the 20 most abundant precursor ions with dynamic exclusion set to 30 s. MS1 scans were acquired at resolution of 120,000 FWHM (at 200 m/z), scan range set to 350–1600 m/z, with an AGC target of 3e6 and a maximum injection time of 100 ms. MS2 scans were acquired at a resolution of 15,000 FWHM (at 200 m/z), scan range set to 200–20000 m/z, with an AGC target of 1e5 and a maximum injection time of 50 ms. Singly charged ions and ions with unassigned charge state were excluded from triggering MS2 events. The normalized collision energy was set to 28%, the mass isolation window was set to 1.4 m/z and one microscan was acquired for each spectrum.

For the human SNU-449 cell and mouse L-dKO tumor tissue experiments, the acquired raw-files were imported into the Progenesis QI software (v2.0, Nonlinear Dynamics Limited), which was used to extract peptide precursor ion intensities across all samples applying the default parameters. The generated mgf-file was searched using MASCOT against either a human database (containing 40724 forward and reverse protein sequences downloaded from Uniprot on 20200417) and 392 commonly observed contaminants or a murine database (containing 34954 forward and reverse protein sequences downloaded from Uniprot on 20200417) and 392 commonly observed contaminants using the following search criteria: full tryptic specificity was required; 3 missed cleavages were allowed; carbamidomethylation (C) was set as fixed modification; oxidation (M) and acetyl (Protein N-term) were applied as variable modifications; mass tolerance of 10 ppm (precursor) and 0.02 Da (fragments). The database search results were filtered using the ion score to set the false discovery rate (FDR) to 1% on the peptide and protein level, respectively. Quantitative analysis results from label-free quantification were processed using the SafeQuant R package v.2.3.2. (PMID:27345528, https://github.com/eahrne/SafeQuant/) to obtain protein relative abundances. This analysis included global data normalization by equalizing the total peak/reporter areas across all LC-MS runs, data imputation using the knn algorithm, summation of peak areas per protein and LC-MS/MS run, followed by calculation of protein abundance ratios. Only isoform specific peptide ion signals were considered for quantification. To meet additional assumptions (normality and homoscedasticity) underlying the use of linear regression models and t-tests, MS-intensity signals were transformed from the linear to the log-scale. The summarized protein expression values were used for statistical testing of between condition differentially abundant peptides using the previously described Perseus software. To visualize the data, we used Perseus’s built-in volcano plot function. Thresholds were set at FDR = 0.05 and S0 = 0.1.

For the human HCC tumor tissue experiment, raw files were searched using Fragpipe (MSFragger-3.4, Philosopher_v4.1.0 against a human database (containing 40748 forward and reverse protein sequences downloaded from Uniprot on 20220222) and 392 commonly observed contaminants using the following search criteria: enzymatic cleavage was set to “stricttrypsin”; 2 missed cleavages were allowed; carbamidomethylation (C) was set as fixed modification; oxidation (M) and acetyl (Protein N-term) were applied as variable modifications; mass tolerance of 20 ppm (precursor) and 20 ppm (fragments). The database search results were to set the false discovery rate (FDR) to 1% on the peptide and protein level, respectively. Quantitative analysis results from label-free quantification were processed using the Perseus software. Intensity data were log_2_ transformed and subsequently filtered for 2 valid values in at least 1 group (samples from leucine-beads and arginine-beads) for the remaining non valid values imputation was performed by normal distribution (width = 0.3/downshift = 3). To visualize the data, we used Perseus’s built-in volcano plot function. Thresholds were set at FDR = 0.05 and S0 = 0.1.

#### Downstream processing of MS data from pull-down experiments

Using the previously described software Perseus, intensity data were log_2_ transformed and subsequently filtered for 2 valid values in at least 1 group (samples from leucine-beads and arginine-beads) for the remaining non valid values imputation was performed by normal distribution (width = 0.3/downshift = 3). To visualize the data we used Perseus’s built-in volcano plot function. Thresholds were set at FDR = 0.05 and S0 = 0.1.

#### Expression of RBM39-FLAG, CASTOR1-FLAG, and eGFP-FLAG in HEK293 cells and anti-FLAG purification

HEK293 cells were seeded in 15 cm plates in DMEM complete medium and transfected at 70–80% confluency with pCMV6-RBM39-FLAG (Cat# RC224584), pCMV6-CASTOR1-FLAG (Cat# RC205868) purchased from Origene (USA) or eGFP-FLAG (serving as control) using jetPRIME from Polyplus (France). Cells were harvested after 48 h in lysis buffer (1% (v/v) IGEPAL-CA630, 2.5 mM MgCl_2_, cOmplete inhibitor cocktail (Roche) and PhosSTOP (Roche), 40 mM HEPES-NaOH pH 7.4) and protein concentration was determined by BCA assay. Expression of RBM39-FLAG, CASTOR1-FLAG, and eGFP-FLAG was controlled by immunoblotting against FLAG tag. 50 μL anti-FLAG beads from Genscript (Netherlands) (Cat# L00432) were equilibrated twice with 1 mL lysis buffer. Binding of 2 mg of protein to equilibrated beads was allowed for 4 h at 4°C and 15 rpm rotation. Beads were washed once with lysis buffer followed by three washes with wash buffer (1% (v/v) IGEPAL-CA630, 2.5 mM MgCl_2_, 500 mM NaCl, cOmplete inhibitor cocktail (Roche) and PhosSTOP (Roche), 40 mM HEPES-NaOH pH 7.4). After complete removal of wash buffer, beads were either used to assess ^3^H-arginine binding (see below) or resuspended in 1X SDS sample buffer and boiled at 95°C for 10 min at 1400 rpm for analysis by immunoblotting.

#### Expression of RBM39-Strep in *E. coli* cells and anti-strep purification

Full-length human *RBM39* was cloned into a pETG-10K vector (gift from Timm Maier) containing a Kanamycin cassette and a C-terminal Strep-tag II (WSHPQFEK) using the gateway method.^69^ Truncation constructs pETG-10K-RBM39(1–244)-C-Strep, pETG-10K-RBM39(143–529)-C-Strep, and pETG-10K -RBM39(245–529)-C-Strep were cloned by SDM using site-specific primers as listed in Table S5. For protein production, chemically competent *E. coli* BL21 (DE3) cells (Thermo Fisher) were transformed according to the heat-shock method. Single colonies were picked and grown in standard LB medium at 37°C and 180 rpm to an OD_600_ of 0.5. Protein expression was induced by the addition of 1 mM IPTG and cells were further grown at 37°C and 180 rpm for 3 h. As background-binding control for the assessment of ^3^H-arginine binding (see below), non-induced, non-transformed *E. coli* BL21 (DE3) cells were grown in parallel, without IPTG addition. Cell pellets were collected by centrifugation (6,000 x*g*, 15 min, 4°C) and stored at −80°C until use. Cell pellets were thawed, resuspended in 3x (v/w) lysis buffer (20 mM HEPES-NaOH, pH 7.4, 500 mM NaCl, cOmplete inhibitor cocktail (Roche)) and lysed by sonication (Hielscher UP200St, 40 W, 30 min, 10 s on, 20 s off). Cell debris was removed by centrifugation (250,000 x*g*, 30 min, 4°C). Protein concentration of the supernatant was determined by BCA assay and analyzed for RBM39-Strep expression by immunoblotting. Equal amounts of protein were incubated with Strep-Tactin Sepharose resin (IBA Lifesciences, 0.5 mg protein lysate/μL resin) equilibrated with high salt buffer (20 mM HEPES-NaOH pH 7.4, 500 mM NaCl) under agitation over night at 4°C. Beads were washed three washes with wash buffer (1% (v/v) IGEPAL-CA630, 2.5 mM MgCl_2_, 500 mM NaCl, cOmplete inhibitor cocktail (Roche) and PhosSTOP (Roche), 40 mM HEPES-NaOH pH 7.4). After complete removal of wash buffer, beads were either used to assess ^3^H-arginine binding (see below) or resuspended in 1X SDS sample buffer and boiled at 95°C for 10 min at 1400 rpm for analysis by immunoblotting.

#### ^3^H-arginine binding to purified RBM39-FLAG and RBM39-Strep

A pair of FLAG beads or Strep-Tactin Sepharose resin bound with RBM39-FLAG (or eGFP-FLAG for control) or RBM39-Strep (or control lysate), respectively (see above), was resuspended in binding buffer (0.1% (v/v) IGEPAL-CA630, 2.5 mM MgCl_2_, 10 mM NaCl, 150 mM KCl, cOmplete inhibitor cocktail (Roche) and PhosSTOP (Roche), 40 mM HEPES-NaOH pH 7.4) containing 10 μM ^3^H-arginine and incubated on ice for 30 min with mild mixing every 5 min. Beads were washed five times with binding buffer in the presence or absence of 100 mM non-radiolabeled arginine and bound ^3^H-arginine was measured with a scintillation counter. Background signals (i.e., counts of bound eGFP-FLAG or control lysate after washes with binding buffer in the presence of 100 mM non-radiolabeled arginine) were subtracted.

#### Proteome of HCC patients

Fresh liver biopsies from 49 HCC were immediately snap-frozen in liquid nitrogen, processed as previously described,^23^^,^^70^ and used for proteomic analysis.^50^ Human HCC biopsies were measured by sequential window acquisition of all theoretical mass spectra (SWATH), in which data-independent acquisition is coupled with spectral library match.^71^ We computed the log_2_-fold-changes of protein abundance between paired tumor and non-tumor tissues for downstream analysis.

#### Transcriptome/RNA-sequencing (RNA-seq) of HCC patients

RNA-seq library prep was performed with 200 ng total RNA using the TruSeq Stranded Total RNA Library Prep Kit with Ribo-Zero Gold (Illumina) according to the manufacturer’s specifications. We computed the log_2_-fold-changes of normalized RSEM gene counts between tumors and the matched non-tumor livers for downstream analysis. RNA-sequencing data^50^ of the human HCCs are available at the European Genome-phenome Archive under accession EGAS00001005074.

#### RNA-seq of cell lines

For RNA-seq of SNU-449 ARG1/AGMAT expressing and control cells, cells were cultured as described above. For RNA-seq of RBM39 depleted and control SNU-449 cells, we combined siRNA-mediated *RBM39* knockdown with indisulam treatment, each as described above. Preparation of samples for RNA-Seq, quality control, and sequencing were performed as previously described.^72^ In brief, the QuantiFluor RNA System (Promega (USA), Cat# E3310) was used to quantify RNA samples fluorometrically. Quality of RNA samples was checked on the TapeStation instrument (Agilent Technologies (USA)) using the High Sensitivity RNA ScreenTape (Agilent, Cat# 5067–5576). Starting from 200 ng of total RNA, library preparation was performed using the TruSeq Stranded mRNA Library Kit (Illumina (USA), Cat# 20020595) and the TruSeq RNA Unique Dual (UD) Indexes (Illumina (USA), Cat# 20022371) with 15 cycles of PCR. Libraries were quality-checked on the Fragment Analyzer (Advanced Analytical (USA)) using the High Sensitivity NGS Fragment Analysis Kit (Advanced Analytical (USA), Cat# DNF-474) revealing excellent quality of libraries (average concentration was 133 ± 11 nmol/L and average library size was 343 ± 14 base pairs). Samples were pooled to equal molarity and quantified by fluorometry using the QuantiFluor ONE double-stranded DNA System (Promega (USA), Cat# E4871). Libraries were sequenced paired-end 51 bases bases (in addition: 8 bases for index 1 and 8 bases for index 2) using the NovaSeq 6000 instrument (Illumina (USA)) and the SP Flow-Cell loaded at a final concentration in Flow Lane of 380 p.m. and including 1% PhiX. Primary data analysis was performed with the Illumina real-time analysis (RTA) version 3.4.4. On average per sample: 39 ± 3 millions pass-filter reads were collected on 1 SP Flow-Cell.

#### Statistical analysis of cell line RNA-seq

Normalized Log_2_ feature counts of RNA-seq analysis were processed using Perseus software (version 1.6.14.0). KEGG pathway annotations were imported using Perseus’s built-in annotation tool. Separation between conditions was visualized with Perseus’s PCA function. Significantly differentially expressed genes were determined and visualized with the volcano plot function. Thresholds were set to FDR = 0.02, S0 = 0.1, and Log_2_ = 0.75 respectively. Hierarchical clustering was performed after z-scoring. Maximum number of clusters was set to 300, iterations to 100 and restarts to 10. The row and tree distances were calculated using Spearman correlation. Pathway enrichment analysis (PWEA) was performed using the hierarchical clustering function with binarized data based on the volcano plot, leaving all parameters at default values. Binarization was performed by defining deregulated as 1, and unchanged as 0 following a randomization by 0.1.

#### Comparison of RNA-seq data of ARG1/AGMAT expressing and RBM39 depleted SNU-449 cells

To compare the two RNA-seq datasets with binarized data, we used the top 2,500 differentially expressed genes from ARG1/AGMAT expressing SNU-449 cells (log_2_ over control expressing SNU-449 cells) defined by p value followed by the absolute LFC threshold of 0.5. The resulting p value cutoff (-Log_10_ p = 1.017) was then applied to the RNA-seq dataset of RBM39 depleted SNU-449 cells (log_2_ over control SNU-449 cells) using again the same LFC threshold of 0.5.

#### Differential alternative splicing analysis

Aligned bam files were generated from fastq files with STAR-aligner (STAR/2.7.9a-GCC-7.3.0-2.30). The R package <NxtIRFcore> was used to generate differential alternative splicing data. The data were filtered using default filters. The function <limma_ASE> was used with default settings to generate statistics for differential ASE (Alternative Splice Elements) analysis with a stringent cut-off (-Log_10_ p = 4).

#### Histopathology and immunohistochemistry

Mouse livers were fixed in 4% (w/v) paraformaldehyde, dehydrated, embedded into paraffin, cut into sections of 4 μm, and placed on SuperFrost slides (Thermo Scientific). Tissue microarray including an independent cohort of 192 HCCs and 79 normal liver samples was cut into sections of 4 μm, and placed on SuperFrost slides (Thermo Scientific).^73^ Immunohistochemistry was performed upon Benchmark immunohistochemistry staining system (Bond, Leica) with Bond polymer refine detection solution for DAB, using ARG1 (HIER citrate buffer pH = 6, 1:2500, Genetex, GTX109242), AGMAT (HIER EDTA buffer pH = 9, 1:100, Sigma, PA5-55311). Immunoreactivity was evaluated by two board-certified experienced pathologists with expertise in gastrointestinal pathology (Caner Ercan and Luigi M. Terraciano). After excluding samples for which the tissue core was absent of had poor staining quality, 58 normal liver and 160 HCC, and 49 normal samples and 142 HCC were available for the analysis of ARG1 and AGMAT in the TMA, respectively.

#### Kaplan-Meier survival curve

RNA sequencing gene expression data including outcomes from 298 hepatocellular carcinomas were obtained from The Cancer Genome Atlas dataset (TCGA, Provisional) via cbioportal (www.cbioportal.org). Downregulation of ARG1 or AGMAT was defined as *Z* score < −0.5.

### Quantification and statistical analysis

The investigators were not blinded to the treatment groups. Data are shown as mean ± SD. Sample numbers are indicated in each figure legend. For mouse experiments, n represents the number of animals, and for cell culture experiments, N indicates the number of independent experiments. To determine the statistical significance paired or unpaired two-tailed Student’s *t* test, multiple t-test, one-way ANOVA, or log rank test were performed using GraphPad Prism 9 Software. A p value of less than 0.05 was considered statistically significant.

## Data Availability

• RNA-seq data have been deposited at GEO (Accession# PRJNA940402). Proteomic (Accession# MSV000091516) and metabolomic (Accession# MSV000092406) data have been deposited at MassIVE (UCSD). All data will be publicly available as of the date of publication. Links and accession numbers are provided in the key resources table. Original immunoblot and microscopy images reported in this paper will be shared by the lead contact upon request. • This paper does not report original code. • Any additional information required to reanalyze the data reported in this paper is available from the lead contact upon request.

## Supplemental information

Table S1. MPWEA of untargeted metabolomics from L-dKO tumor and Ctrl liver tissues, related to Figure 1

Table S2. PWEA using KEGG pathways from RNA-seq of ARG1/AGMAT compared to control expressing SNU-449 cells, related to Figure 3 Metabolic pathways are highlighted in yellow.

Table S3. PWEA of up- and downregulated genes common to ARG1/AGMAT- and RBM39-depleted cells, related to Figure 6

Table S4. Differential alternative splicing analysis upon RBM39 depletion, related to Figure 6

Table S5. qPCR and SDM primer nucleotide sequences, related to STAR Methods
